# Strong correlation between electronic bonding network and critical temperature in hydrogen-based superconductors

**DOI:** 10.1038/s41467-021-25687-0

**Published:** 2021-09-16

**Authors:** Francesco Belli, Trinidad Novoa, J. Contreras-García, Ion Errea

**Affiliations:** 1 Centro de Física de Materiales (CSIC-UPV/EHU), Donostia/San Sebastián, Spain; 2 Fisika Aplikatua Saila, Gipuzkoako Ingeniaritza Eskola, University of the Basque Country (UPV/EHU), Donostia/San Sebastián, Spain; 3 Laboratoire de Chimie Théorique (LCT), Sorbonne Université CNRS, Paris, France; 4 Donostia International Physics Center (DIPC), Donostia/San Sebastián, Spain

**Keywords:** Electronic properties and materials, Superconducting properties and materials

## Abstract

By analyzing structural and electronic properties of more than a hundred predicted hydrogen-based superconductors, we determine that the capacity of creating an electronic bonding network between localized units is key to enhance the critical temperature in hydrogen-based superconductors. We define a magnitude named as the networking value, which correlates with the predicted critical temperature better than any other descriptor analyzed thus far. By classifying the studied compounds according to their bonding nature, we observe that such correlation is bonding-type independent, showing a broad scope and generality. Furthermore, combining the networking value with the hydrogen fraction in the system and the hydrogen contribution to the density of states at the Fermi level, we can predict the critical temperature of hydrogen-based compounds with an accuracy of about 60 K. Such correlation is useful to screen new superconducting compounds and offers a deeper understating of the chemical and physical properties of hydrogen-based superconductors, while setting clear paths for chemically engineering their critical temperatures.

## Abstract (web summary)

Several hydrides have been observed high-*T*_c_ superconductivity under pressure, but a physical-chemical understanding of the properties enhancing *T*_c_ is still lacking. Here, the authors propose a magnitude named as networking value, combined with hydrogen fraction and the contribution of hydrogen to the density of states at Fermi level, can predict *T*_c_ of all hydrogen-based compounds with an accuracy of about 60 K.

## Introduction

The field of hydrogen-based superconductivity has progressed enormously since 1968, when Ashcroft first proposed that pressurized hydrogen may become a high-temperature superconductor due to the high energy of its phonons^1^. While the first discovered superconductors were Th_4_H_15_^2^ in 1970 and PdH^3^ in 1972, with not very promising critical temperatures (*T*_*c*_) of 7.6 and 5 K, respectively, the more recent experimental discoveries at high pressures show that superconductivity on hydrogen-based compounds can span from few Kelvin to room temperature. Few examples of low *T*_*c*_ compounds are the recently synthesized PrH_9_^4^, where *T*_*c*_ = 7 K at 125 GPa, and AlH_3_ where no superconductivity was observed above 4 K despite the predictions^5^. On the other extreme, H_3_S^6^, YH_9_^7,8^, YH_6_^9^, and LaH_10_^10,11^ reach critical temperatures well above 200 K at megabar pressures. In addition, the recent observation of a *T*_*c*_ of 288 K at 267 GPa in a compound formed by sulfur, carbon, and hydrogen^12^ confirms that hydrogen-based superconductors can be room-temperature superconductors. Hydrogen-based compounds are thus the best currently available candidates to reach ambient temperature and pressure superconductivity.

Despite the combination of first principles electron-phonon calculations and crystal structure predictions has been able to anticipate many of the recent outstanding experimental results^13–15^, a simple physical-chemical understanding of the properties enhancing the critical temperatures in hydrogen-based systems is still lacking, hindering the discovery of new compounds with high *T*_*c*_ at low pressures. Indeed, having high-energy phonons is not a guarantee by itself of high-temperature superconductivity. The hundreds of compounds predicted to be superconductors by ab initio crystal structure prediction techniques constitute a rich working dataset to extract conclusions that go beyond this simple idea^13–15^. Among these predictions, the highest *T*_*c*_ values are 300 K for pure metallic hydrogen^16^ and 326 K for the YH_10_ binary compound^17^. Attempts have been made to increase *T*_*c*_ further through ternary compounds, for instance with H_3_S_1−*x*_P_*x*_^18^ and Li_2_MgH_16_^19^. Aiming at extracting useful information from this dataset, two main routes are being explored: on the one hand, machine learning methods^20–22^ are starting to be employed to further increase the list of predicted systems, although the obtained new compounds so far do not beat the already known; on the other hand, additional efforts are being invested into classifying these superconductors using simple footprints based on structural, chemical, and electronic properties^14,21,23^. These studies suggest hydrogen rich systems with highly symmetrical structures and high density of states (DOS) at the Fermi level are the best candidates for high-temperature superconductivity.

Even if these properties are able to suggest good trends, they serve necessary but not sufficient conditions. This ultimately means there are not good optimizers thus far: improving these parameters will not necessarily lead to an improvement of the superconducting critical temperatures. In other words, even if the footprints for a good superconductor are somewhat clear, we cannot yet rely on simple variables to estimate the superconducting temperatures, clarify the reason for such a broad spectrum of *T*_*c*_ values, and, ultimately, chemically engineer better superconductors.

In this work we investigate the chemical, structural, and electronic properties through ab initio methods based on density functional theory (DFT) for a set of 178 hydrogen-based superconductors previously predicted in the literature^13^, including pure hydrogen and binary compounds. Our ultimate goal is to provide a simple understanding of the origin of the high *T*_*c*_ in these compounds. We focus mainly on the electronic and structural properties by means of chemical bonding descriptors, hydrogen–hydrogen distance, electronic charge, and density of states at the Fermi level. We review the impact on the predicted *T*_*c*_ of many of these descriptors, which have been already somewhat studied on a case to case basis in the literature. None of them reveals conclusive. We identify that the electron pairing and delocalization play instead the final role. This takes us to propose a universal descriptor based on the identification of electronic delocalization networks, identified by means of the electron localization function (ELF)^24–28^. We define a simple magnitude, the networking value, which is easily obtained from the calculation of ELF isosurfaces. Such quantity reveals useful to have a first estimate of the superconducting critical temperature without performing electron-phonon coupling calculations. To the best of our knowledge, it is the first time that such a descriptor is proposed in the literature. The networking value could reveal strongly insightful in understanding the physics and chemistry of high-temperature superconductivity and, at the same time, it provides paths for chemically engineering better hydrogen-based superconductors, guiding the quest for high-*T*_*c*_ compounds among the vast possibilities offered by ternary compounds.

## Results

### Chemical composition and bonding categories

In order to provide a comprehensive understanding of the different type of hydrogen-based compounds predicted to exist in the literature, it is convenient to categorize them in families according to the nature of the chemical bonding of the hydrogen atoms in the system. Our classification is guided by the study of the ELF^24–28^ and the atomic charge distribution obtained through the Bader analysis^29–32^ (see Methods for more details). The ultimate goal of this investigation is to better understand what kind of chemical interaction is the most beneficial for superconductivity among these compounds.

**Fig. 1:**
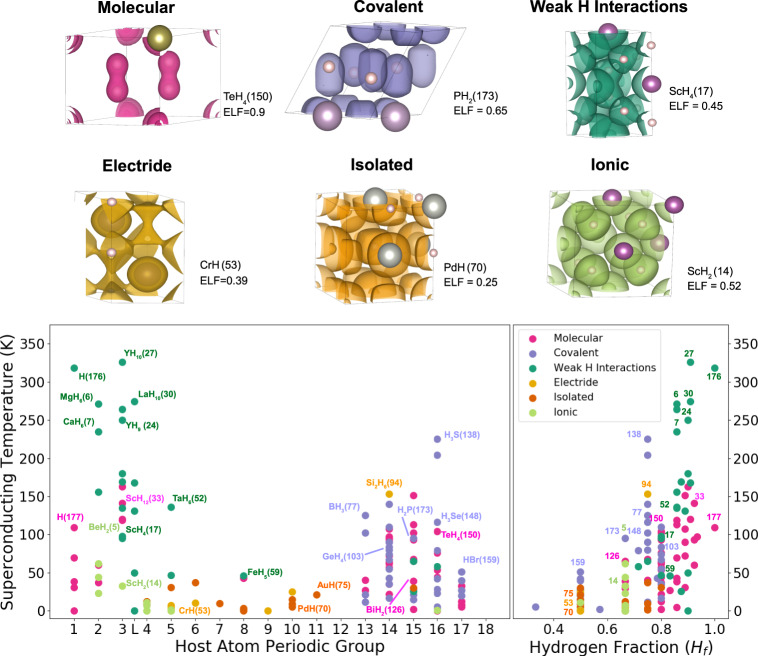
Classification of hydrogen-based superconductors according to their bonding categories. The top panel shows representative systems for the different categories with ELF isosurfaces at different values: TeH_4_ for molecular systems (magenta), PH_2_ for covalent systems (purple), ScH_4_ for systems with weak covalent hydrogen–hydrogen interactions (dark green), CrH for electrides (yellow), PdH for isolated systems (orange), and ScH_2_ for ionic systems (light green). The lower panel shows, respectively, the critical temperature as a function of the host atom periodic group (left panel) and the hydrogen fraction in the compounds (right panel). In the left panel, the name of some compounds is explicitly given together with the index (in parenthesis) given to each compound in Supplementary Table I. In the right panel, and also in Figs. 2 and 4, just the index is given for these compounds for the sake of brevity.

After a thorough analysis of the ELF in our test set, we identify six different families according to the nature of the chemical bonding, namely, molecular systems, covalent systems, systems driven by weak covalent hydrogen–hydrogen interactions, systems with electride behavior, ionic systems, and isolated systems. In each case, the nature of the bonds is identified through the analysis of the ELF saddle points between different atoms. Since bonding properties are mainly local, each system can belong to more than just one family. However, in order to simplify the analysis, we focus on the most dominant feature for each compound. A representative for each family, together with the distribution of the families through the groups of the periodic table and the amount of hydrogen fraction in each case, is shown in Fig. 1. The hydrogen fraction (*H*_*f*_) takes the following form:

1 $${H}_{f}=\frac{{N}_{H}}{{N}_{H}+{N}_{X}},$$

where *N*_*H*_ and *N*_*X*_ are the number of hydrogen and nonhydrogen atoms in the primitive cell, respectively.

The molecular family describes all systems having at least one pair of hydrogen atoms forming a molecule. The latter can be identified through the ELF analysis by locating an isosurface surrounding an isolated molecule at very high values of ELF (see the magenta surface around the hydrogen molecules in TeH_4_ in Fig. 1). A system is chosen to be molecular if an isolated pair of hydrogen atoms appears connected at a value of ELF higher than 0.85, i.e., if the minimum ELF value in between the hydrogen atoms is above 0.85. Systems with molecular behavior appear between groups 1–4 and 13–17. We note that molecular systems tend to have very high values of *H*_*f*_, which reflects that in most cases several molecules exist per host atom. The highest critical temperatures for these molecular compounds have been predicted for ScH_12_(33) and TeH_4_(150), with *T*_*c*_ values around 150 K (the number in parenthesis after a given compound corresponds to the index given for each compound in Supplementary Table I). The critical temperatures of the molecular compounds span from few Kelvin to such high values.

The covalent family is composed of systems where covalent bonds between hydrogen and the host atoms are dominant. Throughout our discussion we shall label the host atom as X, with X ≠ H. For H–X bonds, the covalent character can be identified by an elongation toward the host atom of the ELF isosurface surrounding the hydrogen (i.e., a polarized covalent bond). This is exemplified by the purple surfaces pointing from the H (small spheres) to the P atoms (big spheres) in PH_2_ in Fig. 1. Covalent systems appear for groups 13–17, and are mostly related to the host atom’s *p* type orbital character. Some of the highest critical temperature for these systems have been predicted for H_3_S(138) at 200 K, BH_3_(77) at 125 K, and H_3_Se(148) at 110 K.

The weak covalent hydrogen–hydrogen interaction family is dominated by compounds with predominant weak H–H covalent interactions. The difference with the molecular case is that hydrogen molecules or clusters appear elongated or quasi dissociated. This is illustrated with the dark green surfaces in ScH_4_ in Fig. 1, where bonds between hydrogen atoms appear at much lower values of ELF. From a quantitative point of view we assume that a group of hydrogen atoms is weakly bonded if the ELF at the bond point is within the range [0.4–0.85]. These systems mostly appear between groups 1 to 5. In this bonding type, interactions seem to be purely related to hydrogen atoms whilst the host atoms appear as inert or acting as a chemical precompressor or electron donor. Compounds with this bonding characteristic tend to contain many hydrogen atoms per host, as it happens for the molecular compounds. For compounds with the host atom in a low group of the periodic table, the host atoms valence electrons are donated to the hydrogen atoms resulting in a weakening of the H–H bonds, which translates into an increment of the H–H distance. This family shows the highest predicted critical temperatures, the highest being 326 K for YH_10_(27) and 300 K for metallic hydrogen H(76).

The ionic family is formed by those systems whose hydrogen atoms show an ionic character. This is identified by an isolated proto-spherical ELF isosurfaces surrounding the hydrogen atoms. In addition, for a system to be hereby considered as ionic the mean extra charge per hydrogen atom must be more than 0.5 electrons. This is illustrated by the spherical light green surfaces around hydrogen in ScH_2_ in Fig. 1, where the charge on H is 0.67 electrons. Ionic behavior was observed between groups 2 and 5 of the periodic table, in all cases with low values of *H*_*f*_. The ionic bonding originates from the strong difference in electronegativity between host and hydrogen atoms, which is always increased under pressure^33^. Critical temperatures for these systems are low, with the highest being 45 K for BeH_2_(4).

The electride family contains systems featuring electride behavior, i.e., compounds with electrons localized in the voids. The latter can be identified by isolated pockets of localized electrons in empty space of the crystal as the ones shown for CrH in Fig. 1. From a quantitative viewpoint, electride behavior is characterized in terms of isolated isosurfaces not surrounding any nuclei with ELF maximum values in between 0.35 and 0.7. Note that metallic compounds are also included in this family. Metallic cases also show isolated bubbles of ELF occupying the voids, but their profile is flatter. Given the difficulties to set a quantitative barrier, we have merged them in a unique family. Electrides and metals appear mainly between groups 5 and 10, and are among the systems with the lowest value of *H*_*f*_, reaching a maximum of three hydrogen atoms per host (*H*_*f*_ = 0.75). Critical temperatures for these systems are low, not reaching above 50 K, exception made for the Si_2_H_6_(94) with a *T*_*c*_ of 153 K.

The remaining family includes all the materials featuring extremely weak bonds between hydrogen and host atoms. These systems have been named as isolated and are identified by the lack of any kind of connection of the ELF isosurfaces above 0.25. These systems have low critical temperatures not reaching above 40 K and appear mainly between groups 5 and 12 of the periodic table. They also show a weak capacity of hosting a large number of H atoms per X atom.

Overall, our results highlight that characterizing the bonding type of a solid thanks to these families enables to discard a great number of compounds as potential high-*T*_*c*_ compounds. Covalent interactions, be it weak H–H or X–H are the most favorable for high-temperature superconductivity. This allows to identify the potential interesting combination of elements, especially with respect to the increasing search among ternary compounds. The lowest *T*_*c*_ values appear for electrides and isolated compounds, mostly present between groups 5 and 12, which do not show lots of potential as high-*T*_*c*_ compounds. These families also show the lowest values of *H*_*f*_.

### Hydrogen–hydrogen distance and electronic properties

**Fig. 2:**
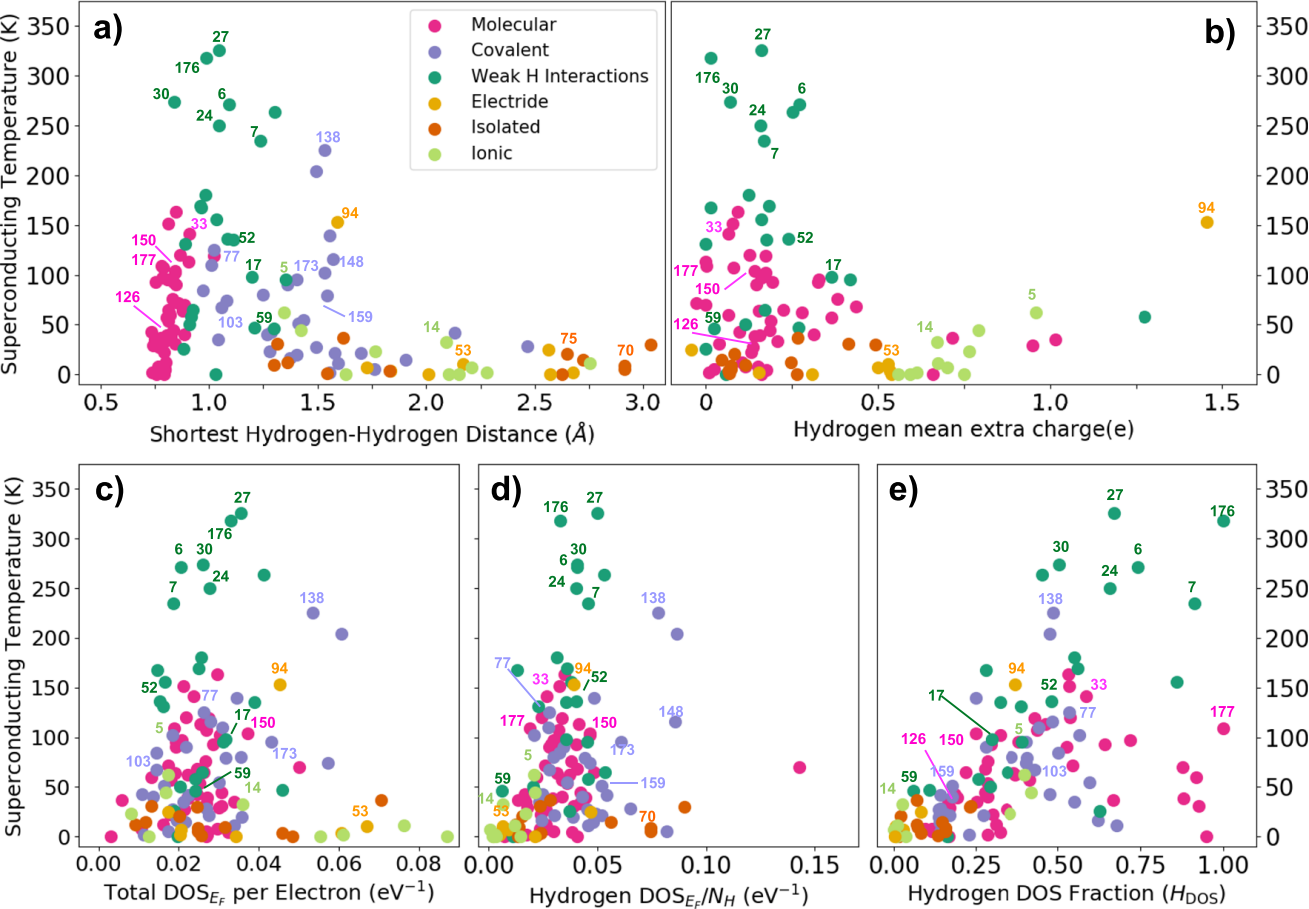
Structural and electronic properties. Panel **a** shows *T*_*c*_ as a function of the shortest hydrogen–hydrogen distance for all compounds. Panel **b** shows *T*_*c*_ as a function of the mean extra electrons per hydrogen atom. Panels **c**–**e**, respectively, show *T*_*c*_ as a function of the total DOS at the Fermi level, the DOS at the Fermi level projected on the hydrogen *s* orbitals per hydrogen, and the fraction of the total DOS at the Fermi level coming from the hydrogen orbitals.

After categorizing the different bonding families of hydrogen-based superconductors, we focus on understanding the trends of the predicted *T*_*c*_ with structural and electronic properties. The results are summarized in Fig. 2. The analysis focuses on the shortest hydrogen–hydrogen distance for each compound, the charge distribution on hydrogen atoms, and the density of states at the Fermi level with the ultimate goal of finding correlations between such descriptors and the critical temperature.

Even if no general trend is observed when plotting *T*_*c*_ as a function of the H–H shortest distance, several conclusions can be drawn. The structural analysis highlights an increment of the superconducting critical temperature with the increase of the shortest hydrogen–hydrogen distance for those systems where the bonding is driven by pure hydrogen interactions, meaning the molecular and weak covalent hydrogen–hydrogen interaction families. For these two families the H–H distance spans from 0.74 Å for systems with *T*_*c*_ below 1 K to a maximum of around 1.35 Å for the compounds with highest critical temperatures. In the region between 0.9 and 1.35 Å lie the currently predicted compounds with the highest superconducting temperatures reaching values as high as 300 K. In other words, elongated H–H interactions promote *T*_*c*_. Interestingly, our analysis highlights that such H–H distance variation is not related to a variation of pressure, meaning that on a broad level, increasing the pressure does not necessarily yield an increment of the bonding distance, so composition rather than pressure would be a more relevant variable to tweak (see Supplementary Table I to see the pressure at which each compound is studied).

**Fig. 3:**
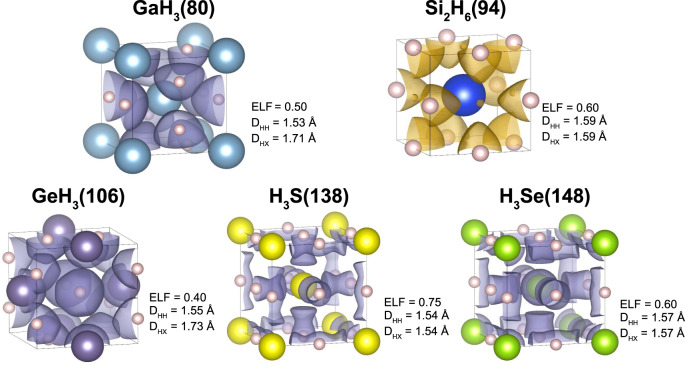
Highly symmetric high-*T*_*c*_ systems. This figure shows five compounds related to the *T*_*c*_ spike around a hydrogen–hydrogen distance of 1.55 Å. The pink spheres refer to hydrogen atoms, while the rest refer to the respective host atoms. An ELF isosurface is depicted in each case. Also the shortest H–H (*D*_HH_) and H–X (*D*_HX_) distances are noted. Most of these compounds belong to the covalent family and show a purple ELF isosurface. The Si_2_H_6_ compound is an electride (note the small ELF bubbles in empty sites) and its ELF isosurface is shown in orange.

The shortest hydrogen–hydrogen distance for the covalent family spans between 1 and 2.5 Å. Low-symmetry covalent systems appear between 1 and 1.45 Å where the short H–H distance is due to interhydrogen bonds appearing beside the dominant hydrogen-host bonds. The highest reported superconducting temperature for these systems is about 135 K for BH_3_(77). Interestingly, at around 1.55 Å the covalent family shows a sharp spike in the predicted *T*_*c*_ through systems sharing linear *X* = *H* = *X* bonds originating through the host *p* orbitals, a $\bar{3}$m point group, a value of *H*_*f*_ equal to 0.75, and a lack of direct hydrogen–hydrogen bonds. Here lay systems as H_3_S(138), H_3_Se(148), GaH_3_(80), and GeH_3_(106). Interestingly, the Si_2_H_6_(94) electride also shares these features even if the bonding nature is slightly different. All these compounds are shown in Fig. 3 with a representative ELF isosurface. For H–H distances above ~1.55 Å a sharp drop in *T*_*c*_ appears, with systems not reaching above 50 K. This zone lacks direct hydrogen–hydrogen bonds and features systems with a low percentage of hydrogen, with mostly isolated, electride, and ionic behavior. Hence, covalent elongated bonds, H–H or H–X, with high *H*_*f*_ seem to be the best candidates to increase *T*_*c*_.

Panel b of Fig. 2 shows the mean extra electron per hydrogen atom obtained through the analysis of the Bader charge (see Methods). The covalent family has been excluded from this panel due to the unreliability of the Bader anlysis, which arises from the difficulties in assigning shared electrons in the H–X bonds to each atom. We estimate the mean extra electron per hydrogen atom ($\bar{\rho }$) as

2 $$\bar{\rho }=\frac{\mathop{\sum }\nolimits_{i = 1}^{{N}_{H}}{Q}_{i}-{N}_{H}}{{N}_{H}},$$

where *Q*_*i*_ is the number of electrons assigned to the *i*th hydrogen atom by the Bader analysis, while *N*_*H*_ is the total number of hydrogen atoms in the primitive cell.

Overall hydrogen atoms tend to gain electrons due to their higher electronegativity with respect to other atoms in the cell. We observe that the highest *T*_*c*_ values are associated with small charge transfers, i.e., from 0 to 0.25 extra electrons per hydrogen atom. As the extra charge increases, *T*_*c*_ drops sharply below 50 K, exception made for the BeH_2_(5), compound belonging to the ionic family with *T*_*c*_ = 97 K, and Si_2_H_6_(94), with *T*_*c*_ = 153 K and part of the electride family.

It is often mentioned in the literature that an increment of (negative) charge on the hydrogen atom leads to a weakening of the hydrogen–hydrogen bonds^19,34–36^. Our results show that both molecular and weak covalent hydrogen–hydrogen interaction families show non-negligible extra electrons on the hydrogen atoms. We also observe that the extra charge on the hydrogen is responsible for a slight increment of the H–H distance for the weak covalent hydrogen–hydrogen interaction family. In fact, compounds within this family with a shorter H–H distance tend to have less extra electrons per hydrogen atom. The maximum extra electron per hydrogen among this family is around 0.5, a value that increases up to 1 in the molecular systems.

Panels c, d, and e in Fig. 2 report the results for the DOS analysis. The total DOS per electron at the Fermi level shown in panel c of Fig. 2 shows a sharp increment in the highest superconducting temperatures for a DOS value of around 0.015 *eV*^−1^. However, for such values of the DOS, compounds with very low *T*_*c*_ can still be found, making the total DOS at the Fermi level not a good descriptor of high-*T*_*c*_ compounds as it has already been suggested in the literature^37^. This is somewhat unexpected as the electron-phonon coupling constant (*λ*) is proportional to the total DOS at the Fermi level.

Considering that H atoms due to their light mass are responsible for the large values of *T*_*c*_ in these compounds, we analyze the projection of the DOS at the Fermi level onto hydrogen atoms (see panel d of Fig. 2). However, the trends are similar to those obtained for the total DOS. Our results suggest thus that the key for high superconductivity is not strictly related to the value of the DOS. Instead, the fraction of active hydrogen atoms at the Fermi energy (*H*_DOS_) reported in panel e seems to be more relevant. This quantity is obtained dividing the contribution to the DOS at the Fermi energy coming from the hydrogen orbitals by the total DOS at the Fermi level. In agreement with our previous findings, only molecular and weak covalent hydrogen–hydrogen interaction families are able to reach high amounts of DOS coming from the hydrogen active atoms. This is directly related to the fact that the H–H bonds in those systems have a great contribution to the HOMO and LUMO. Nevertheless, false positives exist, specially among molecular compounds, where the *T*_*c*_ appears very low despite the large contribution of hydrogen states to the Fermi surface. In fact, the DOS misses the information on how electrons are coupled with the lattice vibrations. Systems with very high values of the DOS at the Fermi energy but with very low electron-phonon coupling will not exhibit high superconducting critical temperatures.

### Networking through the ELF

In order to screen new superconductors and guide the quest for new superconducting compounds, we need to look for an easily computable variable that will tell us not only about the interatomic bonding properties, but also how prone the system is to electron-phonon coupling. Even if some trends can be observed as discussed above, the descriptors analyzed so far in Figs. 1 and 2 are unable to capture when electrons couple more strongly to lattice vibrations and, thus, do not correlate well with *T*_*c*_. In this section we propose a new observable based on the study of electron (de)localization that, instead, correlates well with the predicted superconducting critical temperature.

The ELF is a function suited to analyze the degree of electronic localization as high value isosurfaces of the ELF reveal regions in space where electrons localize. In fact, for isosurfaces with values close to 1 the electrons are localized generally on atomic sites, and they start to delocalize toward neighbors and form bonds as the ELF value decreases (see Methods). In order to analyze this delocalization on a crystal size scale, we define the networking value *ϕ* as the highest value of the ELF that creates an isosurface spanning through the whole crystal in all three Cartesian directions. The *ϕ* value can thus be easily extracted by calculating the ELF and determining at which value a crystal sized isosurface is created when lowering the ELF value from 1. This isosurface encloses most of the atoms in the crystal, but not necessarily all. The ELF saddle points reveal crucial for the determination of the networking value, especially for hydrogen-based compounds (see Methods and Supplementary Figs. 1–4 for some examples on the determination of *ϕ*). For these systems, where hydrogen–hydrogen bonds are dominant, the ELF saddle points identify the weak or strong interatomic bonds that pave the crystal sized electronic localization network. Thus, for the determination of the networking value it is sufficient to identify the ensemble of ELF saddle points at the highest value of ELF able to bridge the gap between different atoms and create the 3D network.

**Fig. 4:**
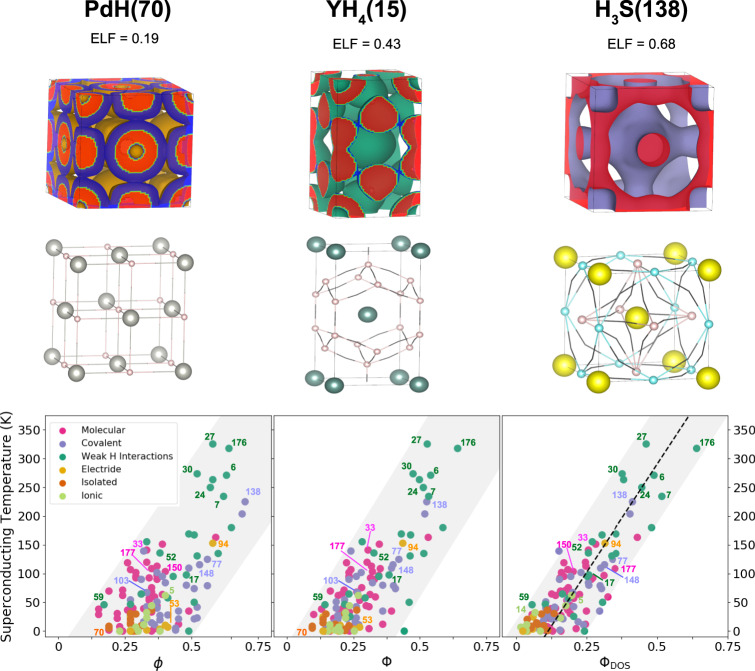
Networking value. The upper panel shows the ELF isosurface and the three dimensional network spanning through all the crystal, which is formed by the ELF saddle points and the atoms, associated with the *ϕ* value for PdH(70), YH_4_(15), and H_3_S(138). The networking value is given for each case. The bottom panels show the critical temperature *T*_*c*_ as a function of the networking value *ϕ* (left), the networking value multiplied by *H*_*f*_ (center), Φ = *ϕH*_*f*_, and the networking value multiplied by *H*_*f*_ and the cubic root of the fraction of the DOS at the Fermi energy coming from the hydrogen atoms *H*_DOS_, ${{{\Phi }}}_{{{{{{{{\rm{DOS}}}}}}}}}=\phi {H}_{f}\root 3 \of {{H}_{{{{{{{{\rm{DOS}}}}}}}}}}$ (right). The dotted line in the last panel represents a fit for which *T*_*c*_ = (750Φ_DOS_ − 85) K which is able to estimate *T*_*c*_ within 60 K.

In Fig. 4 we provide the ELF isosurface related to the *ϕ* value as well as the network created by the saddle points for this ELF value for PdH(70), YH_4_(15), and H_3_S(138). These three cases are related to the most common types of networks identified during the analysis: isolated, weak covalent hydrogen–hydrogen interaction, and covalent families, respectively. In PdH(70) the network includes both Pd and H atoms. The isolated behavior of the atoms makes the ELF bubbles around the atoms network at very low values of the ELF, 0.19. For YH_4_(15) the highest 3D connecting network appears at ELF = 0.43, and is constructed only by hydrogen atoms showing a weak covalent interaction. This is one of the cases in which the 3D network subsisting at the highest value of ELF does not include all the atoms in the unit cell. For the case of H_3_S(138), two interlaced networks appear at ELF = 0.68 due to its high symmetry, which is supported by the *H* = *S* covalent bonds. Another type of network is the one arising from the electride systems, where the connection appears through the isolated pockets of charge in the empty zones of the unit cell.

Interestingly, the networking value correlates rather well with *T*_*c*_ as shown in Fig. 4, clearly much better than any other descriptor based on the structure or the electronic properties studied so far in the literature and in Figs. 1 and 2. We attribute this positive correlation to the capacity of this descriptor to somehow measure how the lattice vibrations affect the electronic cloud. This is not surprising, because if atoms are connected among them with highly localized electrons, phonon vibrations are prone to affect more electrons. The fact that the network is required to span through all the crystal seems also reasonable, as all, or at least many, phonon modes are expected to affect the electronic cloud in this case.

The positive correlation between the networking value and *T*_*c*_ is universal as it holds for all bonding families. This is not surprising as bonding families are determined by localized electrons around atomic cores, while the networking value is related instead to delocalized electrons that bridge the space between locally bonded units. The importance of the degree of electronic localization at the saddle points of the ELF that join locally bonded units seems to be supported by the calculation of the ELF with a model BCS superconducting wavefunction^38,39^ for a diatomic system (see Supplementary Note I). According to the model, irrespective of the value of the superconducting gap, higher networking values also favor higher networking values in superconducting electrons, which we expect to lead to higher electron-phonon coupling. This would explain why DFT calculations of electronic properties such as the ELF are able to anticipate superconductor properties.

As seen in Fig. 4, an improvement in the correlation is obtained by multiplying *ϕ* by the hydrogen fraction *H*_*f*_ of the compound:

3 $${{\Phi }}=\phi {H}_{f}.$$

An explanation for such improvement is that the hydrogen fraction is a rough estimation of the multiplicity of hydrogen bonds. Systems with few H atoms will tend to form less bonds in which H atoms participate. This is in contrast with hydrogen rich systems with an incredible number of bonds in which H atoms participate. A further improvement can be obtained by adding a correction coming from the DOS by defining

4 $${{{\Phi }}}_{{{{{{{{\rm{DOS}}}}}}}}}=\phi {H}_{f}\root 3 \of {{H}_{{{{{{{{\rm{DOS}}}}}}}}}},$$

where *H*_DOS_ is the hydrogen fraction of the total DOS at the Fermi energy. The introduction of this quantity is able to screen out all those systems that have a high value of Φ but a low contribution of H atoms to the DOS, i.e., all systems that present good bonding properties for superconductivity but lack active electrons at the Fermi level to host it. In fact, the networking value does not correlate at all with the DOS at the Fermi level, not even with the DOS coming from H atoms, underlining that these two descriptors are measuring different quantities (see Supplementary Fig. 5). The networking value offers for the first time an adimensional magnitude that shows a striking correlation with *T*_*c*_, which is valid to estimate the superconducting critical temperature in hydrogen-based superconductors. In fact, the superconducting critical temperature of these systems can be predicted, within 60 K, following the *T*_*c*_ = (750Φ_DOS_ − 85) K equation.

**Fig. 5:**
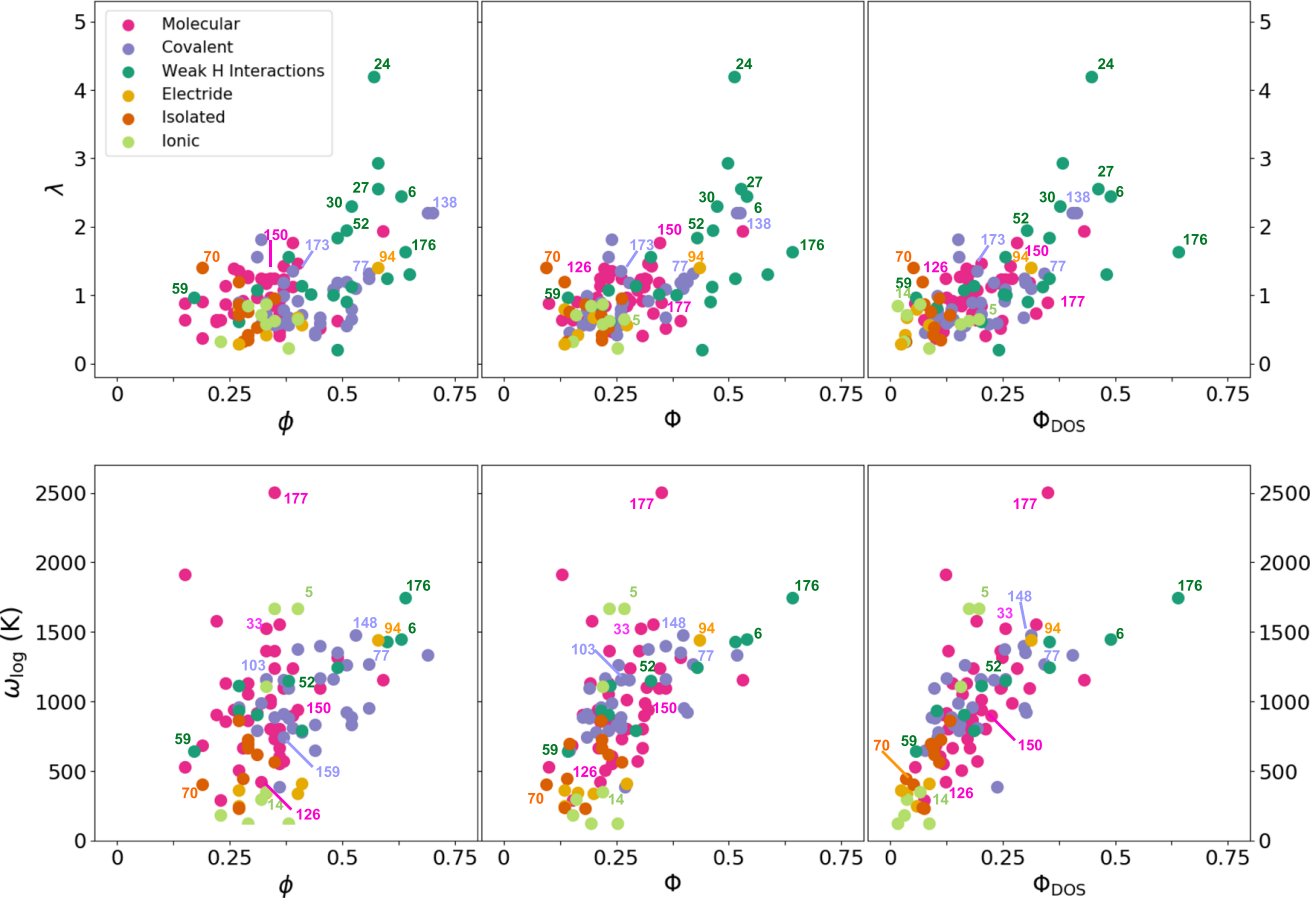
Networking value with respect to *λ* and ${\omega }_{{{{{{{{\rm{log}}}}}}}}}$. The figure shows, respectively, *λ* and ${\omega }_{{{{{{{{\rm{log}}}}}}}}}$ as a function of the networking value (*ϕ*), the reduced networking value (Φ), and the reduced networking value corrected by the hydrogen contribution to the DOS at the Fermi energy (Φ_DOS_).

It is illustrative to look at the simple McMillan equation of the critical temperature

5 $${T}_{c}=\frac{{\omega }_{{{{{{{{\rm{log}}}}}}}}}}{1.2}\exp \left[-\frac{1.04(1+\lambda )}{\lambda -{\mu }^{* }(1+0.62\lambda )}\right],$$

where μ^*^ is the so-called Coulomb pseudopotential, in order to understand if the correlation found with the networking values is assigned to the electron-phonon coupling constant *λ* or to the logarithmic average phonon frequency ${\omega }_{{{{{{{{\rm{log}}}}}}}}}$. As shown in Fig. 5 both *λ* and ${\omega }_{{{{{{{{\rm{log}}}}}}}}}$ correlate with *ϕ*, Φ, and Φ_DOS_. This confirms that the networking value is able to capture how prone electrons are to couple to phonons. Also, the average strength of the bonds, which affects phonon frequencies. However, the correlation found for both *λ* and ${\omega }_{{{{{{{{\rm{log}}}}}}}}}$ is worse than the one found for *T*_*c*_. The reason is that many of the predictions in our dataset are obtained in materials with rather low phonon frequencies, close to structural instabilities. In this regime, while *λ* soars, ${\omega }_{{{{{{{{\rm{log}}}}}}}}}$ is suppressed, which in the end preserves the correlation with *T*_*c*_ also in these cases.

## Discussion

After the large analysis presented in this work, we can conclude that the highest critical temperatures are achieved in the molecular, covalent, and weak covalent hydrogen–hydrogen interaction families. These three families are different expressions of covalent bonds, where electrons are strongly localized. The highest critical temperatures appear between groups 1 and 5 of the periodic table, where bonds are mainly driven by covalent hydrogen–hydrogen interactions, and 13 and 16, where covalent bonds are predominantly between hydrogen and host atoms. It seems that these covalent compounds reach their highest *T*_*c*_ values for systems with large symmetry and hydrogen-host bonds at a distance of about 1.55 Å, without direct hydrogen–hydrogen bonding.

It is important to remark that our work shows how the molecular family transitions towards the weak covalent hydrogen–hydrogen interaction one, with an associated increase in *T*_*c*_. The transition is smooth, starting from low *T*_*c*_ systems with only hydrogen molecules, such as H_4_I(164), going then through a mixed phase where molecules expand and intermolecular H–H interactions start to be present as for ScH_9_(31) and ScH_7_(34), to finally transition towards a full weak interacting behavior with no molecules as found for YH_10_(27) and LaH_10_(30), which have the largest networking value and *T*_*c*_. From our (de)localization analysis, these systems show localized electrons (high ELF maxima), but with a high probability of being coupled (high ELF saddle points), as captured by the networking value. This suggests that stretching hydrogen molecules is beneficial for superconductivity. The same conclusion is reached looking at the two pure hydrogen phases studied here (systems 176 and 177). The hydrogen phase ascribed to the weak covalent hydrogen–hydrogen interaction behavior (176) shows a far higher *T*_*c*_ of around 300 K compared to the purely molecular phase (177), with *T*_*c*_ = 109 K. Therefore, stretching hydrogen molecules by chemical or mechanical means in systems containing many H_2_ units seems a very promising path to discovering new high-*T*_*c*_ compounds. This seems to put in context the extraordinary prediction of a critical temperature of 473 K in Li_2_MgH_16_^19^, where doping a molecular MgH_16_ compound with Li brakes the molecular units, transforming the system into one with weak covalent hydrogen–hydrogen interactions.

The networking value *ϕ* defined here is able to capture effectively how sensitive the electronic cloud is on average to lattice vibrations, and, consequently, correlates well with *T*_*c*_. The networking value improves all other structural or electronic descriptors previously studied^14,21,23^. As extracting *ϕ* simply requires the analysis of ELF isosurfaces, which can be easily obtained post-processing DFT ground state calculations, it offers a simple way of screening hydrogen-based superconductors, as well as showing the correct directions to chemically engineering better hydrogen-based superconductors. It was also noticed that whenever the variation of *T*_*c*_ with pressure was related to a general structural deformation, the value of *ϕ* was also able to correctly capture the pressure effects on *T*_*c*_. Interestingly, as the definition of *ϕ* is completely general, not limited to the presence of hydrogen in the system, it could potentially be used to estimate the *T*_*c*_ of all phonon-mediated superconductors. Eventually, it may be also worth studying it in unconventional superconductors, as *ϕ* is only based on the analysis of the electronic cloud and correlations could also appear.

As a final word, we would like to underline that the superconducting critical temperatures used to find correlations are extracted directly from the literature, without being recalculated (see Supplementary Table I to check the reference from which the *T*_*c*_ value was taken for each case). All the *T*_*c*_ values were obtained by first principles DFT calculations, but at different levels of theory, for instance, for the estimation of the critical temperature. Indeed, for the highest critical temperature systems, the value of *T*_*c*_ can vary up to 40 K depending on the choice of μ^*^. The wide gray area in Fig. 4 could be the result of such inconsistencies. In addition, most of these *T*_*c*_ values have been obtained assuming that the ground state structure is the one given by the minimum of the Born-Oppenheimer energy surface (classical approximation) and that lattice vibrations can be described within the harmonic approximation around these positions. However, in hydrogen-based superconductors, recent calculations have shown that the crystal structure can be largely modified by ionic quantum effects and that anharmonicity strongly renormalizes the obtained harmonic phonon spectra, which can strongly impact the predicted *T*_*c*_^16,17,40–45^. These effects could produce a narrower correlation between *T*_*c*_ and *ϕ* by introducing variations due to corrections on the structure and *T*_*c*_.

## Methods

### DFT calculations

We carry out our analysis on a sample of 178 compounds containing hydrogen that had been previously predicted to be superconductors in the literature. Most of the chosen compounds were those summarized in ref. ^13^, which had been analyzed before in the literature^13,16,34,35,43,44^^,^^46–129^. For each of those compounds we have performed a classical relaxation of the structure at the given reported pressure with DFT at the Born-Oppenheimer minimum position and then we have calculated the electronic properties. Due to the immense work required, we did not perform the *T*_*c*_ calculations, but took the values predicted in the literature. Among the 178 compounds, 43 were discarded due to lack of information on the atomic structure, which made impossible their analysis. Supplementary Table I reports a list of the compounds considered in this analysis, as well as from which reference the *T*_*c*_ value was taken.

All DFT calculations were performed with the plane-wave Quantum ESPRESSO (QE) package^130,131^. The exchange correlation potential was approximated with the Perdew-Burke-Ernzerhof parametrization^132^. At least the first few upper core orbitals were included in the pseudopotential for the host atom. The cutoff for the wavefunctions and the density were, respectively, 70 and 700 Ry. Integrations over the Brillouin zone were performed with the Methfessel-Paxton smearing technique^133^, with a 0.02 Ry broadening. These integrations were performed with dense **k** point grids, where a volume of 0.001 Å^−3^ was occupied per **k** point in the Brillouin zone for the self-consistent calculation and a volume of 0.0002 Å^−3^ for the non self-consistent calculation. The electronic properties such as the ELF, the DOS, and the charge distribution were calculated for each system using the QE post-processing tools through the results obtained for the non self-consistent calculations.

### Bader charges

There have been numerous approaches in order to determine the charge associated to an atom in a molecule. Probably, one of the most useful in solid state is that derived from the electron density, introduced by Bader and coworkers in what is called the Quantum Theory of Atoms in Molecules^134^. In an ordinary solid, the electron density has its maxima (cusps) at the nuclei and decays exponentially as the electron density moves away from the nuclei. The resulting topology looks like an assemblage of mountains, each of which is identified as an atom. The zero gradient surface around these maxima are well defined surfaces that lead to atoms as nonoverlapping units. This allows determining their charge by mere integration of the electron density within their associated region of space. Since these regions are nonoverlapping these charges have the advantageous property of being additive.

### The electron localization function

The electron localization function (ELF) was developed by Becke and Edgecombe in 1990^135^ for the analysis in real space of electron localization, and later on reinterpreted by Savin^136^ in terms of the Pauli kinetic energy density (*t*_*p*_) corrected by the homogeneous electron gas kinetic energy density (*t*_HEG_):

6 $$\chi =\frac{1}{1+{({t}_{p}/{t}_{{{{{{{{\rm{HEG}}}}}}}}})}^{2}}.$$

*χ* is then mapped to run from 0 to a maximum value of 1:

7 $$\,{{\mbox{ELF}}}\,=\frac{1}{1+{\chi }^{2}}.$$

Values close to 1 appear in those places where electrons are localized. Hence, maxima appear in the bonds (as well as in cores and lone pairs). A molecule such as N_2_ will feature a maximum in the middle of the internitrogen distance associated with the N–N bond and separated from the N cores. It should be noted that hydrogen constitutes a peculiar case. Since a hydrogen molecule only has two electrons, a maximum does not appear for the H–H bond, but rather a surface with very high ELF that encapsulates the H_2_ molecule.

It is easy to see from Eq. (7) that the value ELF = 0.5 is associated with the distribution in a homogeneous electron gas of the same density as the point of study. Indeed, metals are characterized by very flat ELF profiles slightly deviating from 0.5.

Whereas the maxima of ELF provide a measure of how localized electrons are, its value in between these maxima characterize delocalization in between these regions, i.e., how easy it is for electrons to go from one localized unit to another^137^.

In order to understand the expected ELF behavior in our systems we have constructed a simple model formed by two Gaussians centered at 0 and 2*R*_0_, which represent atomic cores, and a diffuse function representing a metallic state. In the case of the superconducting state, the diffuse function has been substituted by a real space representation of the Cooper pair. This wavefunction has been obtained from the Kadin wavefunction for a BCS Cooper pair^38,39^ using a local density approximation (see Supporting Information).

### The networking value

To obtain the networking value (*ϕ*) the ELF values in the unit cell were calculated over a cubic grid of 125 × 10^6^ points for each system. Through the use of the program *Critic2*^138,139^ the position of the various ELF saddle points, their ELF values, and the atoms forming the bond to which the saddle points were related were located. Successively, a subset of ELF saddle points was isolated starting from an ELF threshold value close to 1. Such subset of ELF saddle points was visualized together with the interatomic bonds related to the saddle points. The subset of ELF saddle points was then progressively expanded by lowering the ELF threshold until reaching a value for which a 3D network spanning through the entire cell was identified. Such value of ELF defined the *ϕ* for the compound. Few examples for the identification procedure and the appearance of the networks are provided in the Supplementary Figs. 1–4. Note that an automated code for determining the networking value is available at https://www.lct.jussieu.fr/pagesperso/contrera/tcestime/index.html.

## Supplementary information

Supplementary Information

## Data Availability

The list of compounds utilized in this work are summarized in Supplementary Table I. Additional data used in this study are available in the Figshare database under accession codes 10.6084/m9.figshare.15105831. Further data or details are available from the corresponding author upon reasonable request.

## Code availability

Quantum ESPRESSO is an open-source suite of computational tools available at https://www.quantum-espresso.org. *Critic2* is distributed under the GNU/GPL v3 license. The code for calculating the networking value is available at https://www.lct.jussieu.fr/pagesperso/contrera/tcestime/index.html.
