# Supplementary material for: Strong correlation between electronic bonding network and critical temperature in hydrogen-based superconductors
Source: Nat Commun. 2021 Sep 16;12:5381. doi: 10.1038/s41467-021-25687-0 (PMC8446067; doi:10.1038/s41467-021-25687-0)
Supplement: Supplementary file 1 — Supplementary Information [file 41467_2021_25687_MOESM1_ESM.pdf]

Supplementary Information:
Strong correlation between electronic bonding network and critical temperature in hydrogen-based superconductors

Francesco Belli,[1,2] Trinidad Novoa,[3] J. Contreras-Garcia,[3] and Ion Errea*[1,2,4]

[1] *Centro de Física de Materiales (CSIC-UPV/EHU), Manuel de Lardizabal Pasealekua 5, 20018 Donostia/San Sebastián, Spain*
[2] *Fisika Aplikatua Saila, Gipuzkoako Ingeniaritza Eskola, University of the Basque Country (UPV/EHU), Europa Plaza 1, 20018 Donostia/San Sebastián, Spain*
[3] *Laboratoire de Chimie Théorique (LCT), Sorbonne Université CNRS, 75005 Paris (France)*
[4] *Donostia International Physics Center (DIPC), Manuel de Lardizabal Pasealekua 4, 20018 Donostia/San Sebastián, Spain*
**email: ion.errea@ehu.eus*

CONTENTS

Network Examples 1

Density of States 7

Supplementary Note I:
Model superconducting electron localization function 8

Supplementary Table 10

References 13

NETWORK EXAMPLES

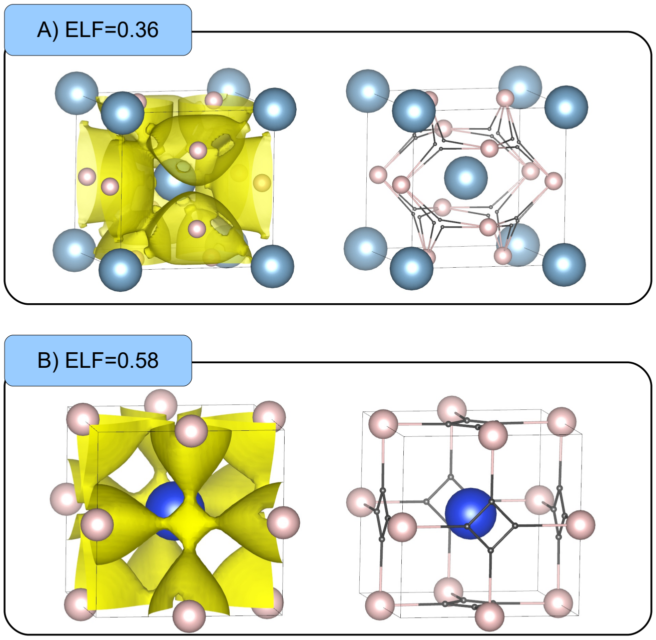

Figure 1. **Examples of Networks arising from Covalent and Electride bonds.** The figure shows an example of a network arising from covalent bonds as in A) for AlH_3 (78), with $\phi = 0.36$, and an example of a network arising from an electride bonding pattern as in B) for Si_2H_6 (94), with $\phi = 0.58$.

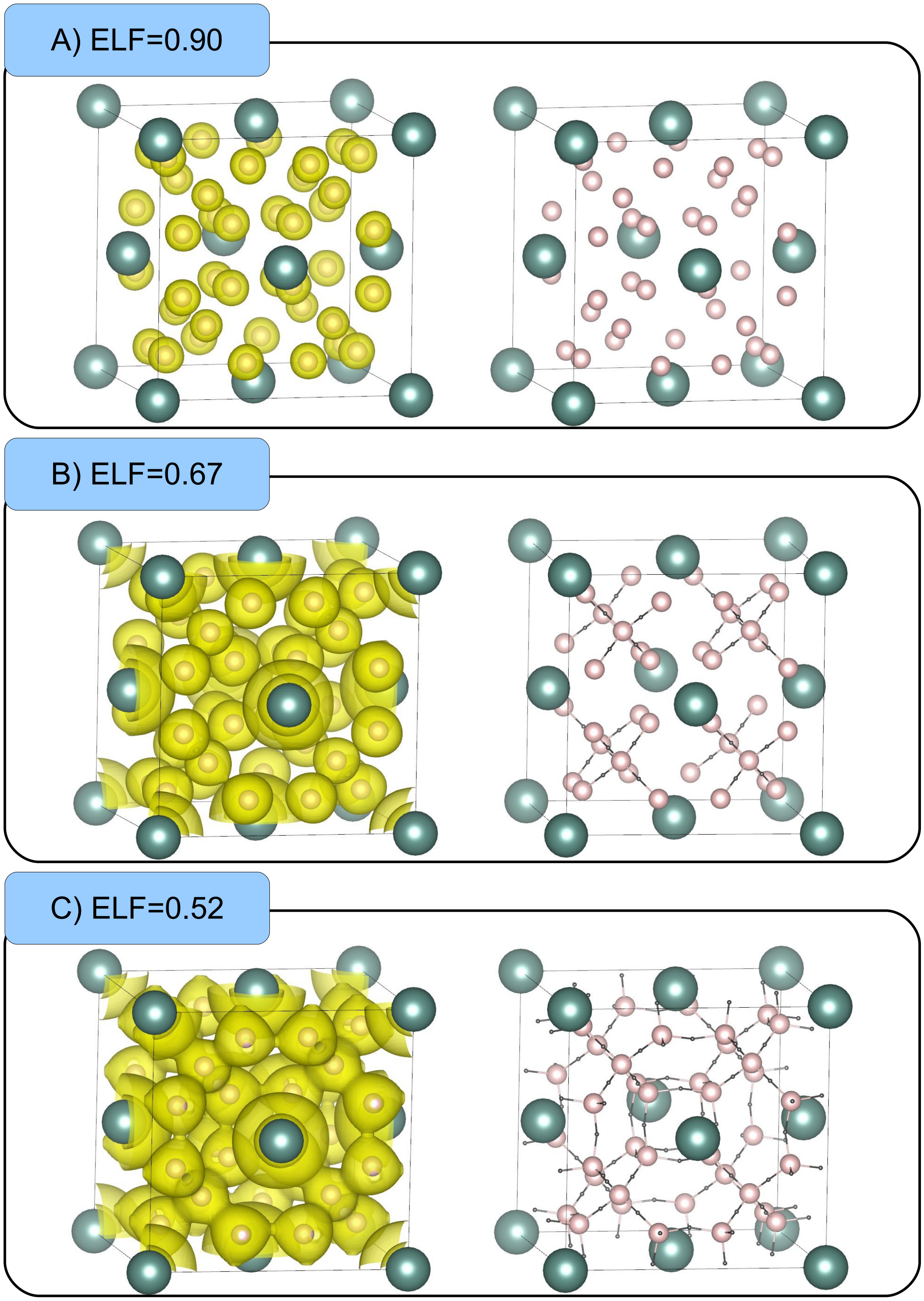

Figure 2. **Example of construction of Network 1.** The figure shows the steps for the construction of the network for LaH_{10} (30). On the left side are visualized the ELF isosurfaces, while on the right side are visualized the network patterns identified through the selection of the saddle points (small black dots) related to the same and higher value of ELF in respect to the ELF threshold in the blue box. In step A) no bond is formed. In step B) few isolated bonding patterns appear. In step C) the highest 3D network spanning through the entire cell is created. The resulting network is constructed by all the hydrogen atoms.

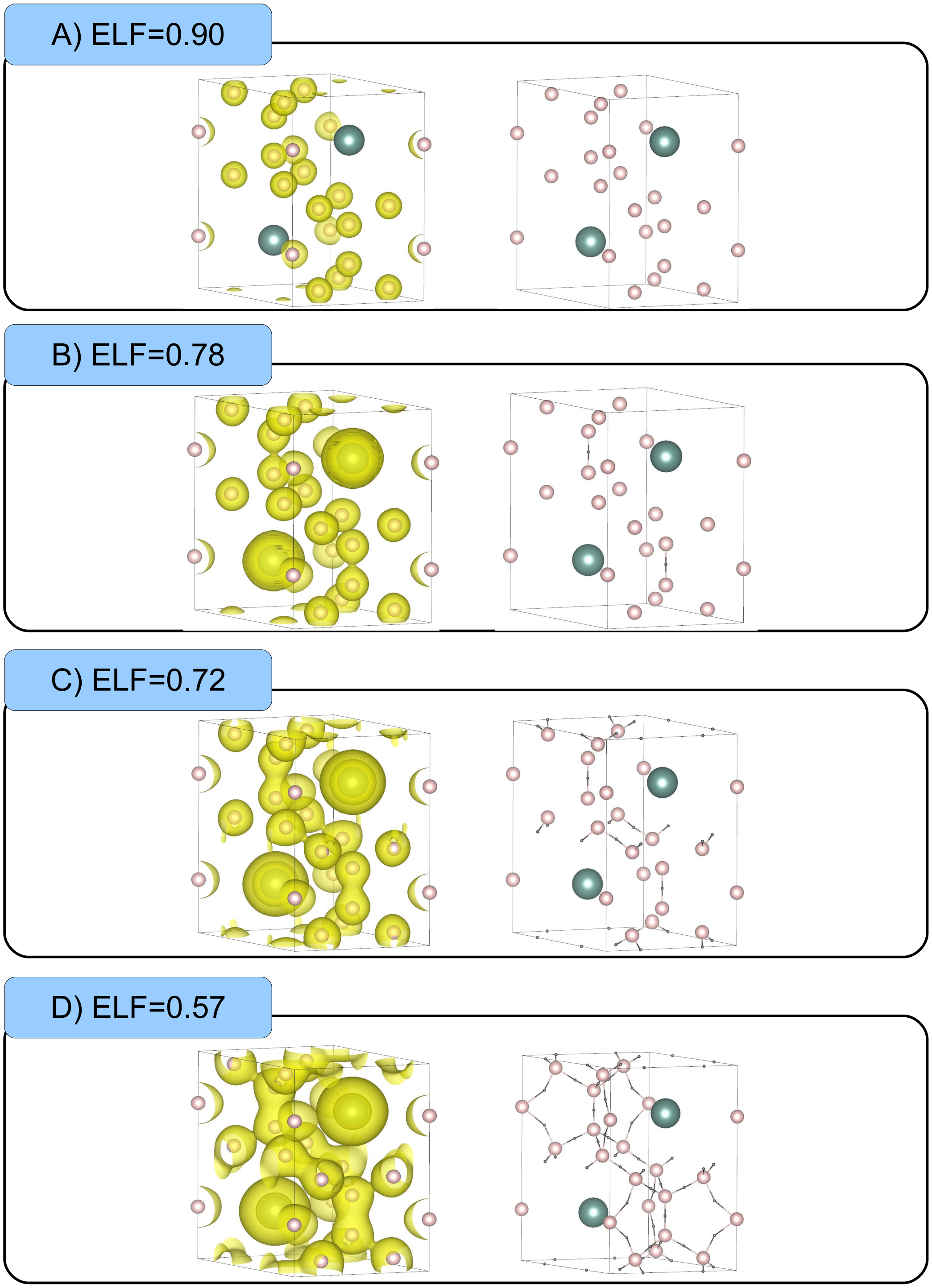

Figure 3. **Example of construction of Network 2.** The figure shows the steps for the construction of the network for YH_9 (24). On the left side are visualized the ELF isosurfaces, while on the right side are visualized the network patterns identified through the selection of the saddle points (small black dots) related to the same and higher value of ELF in respect to the ELF threshold in the blue box. In step A) no bond is formed. In step B) and C) few isolated bonding patterns appear. In step D) the highest 3D network spanning through the entire cell is created. The resulting network is constructed by a subset of hydrogen atoms.

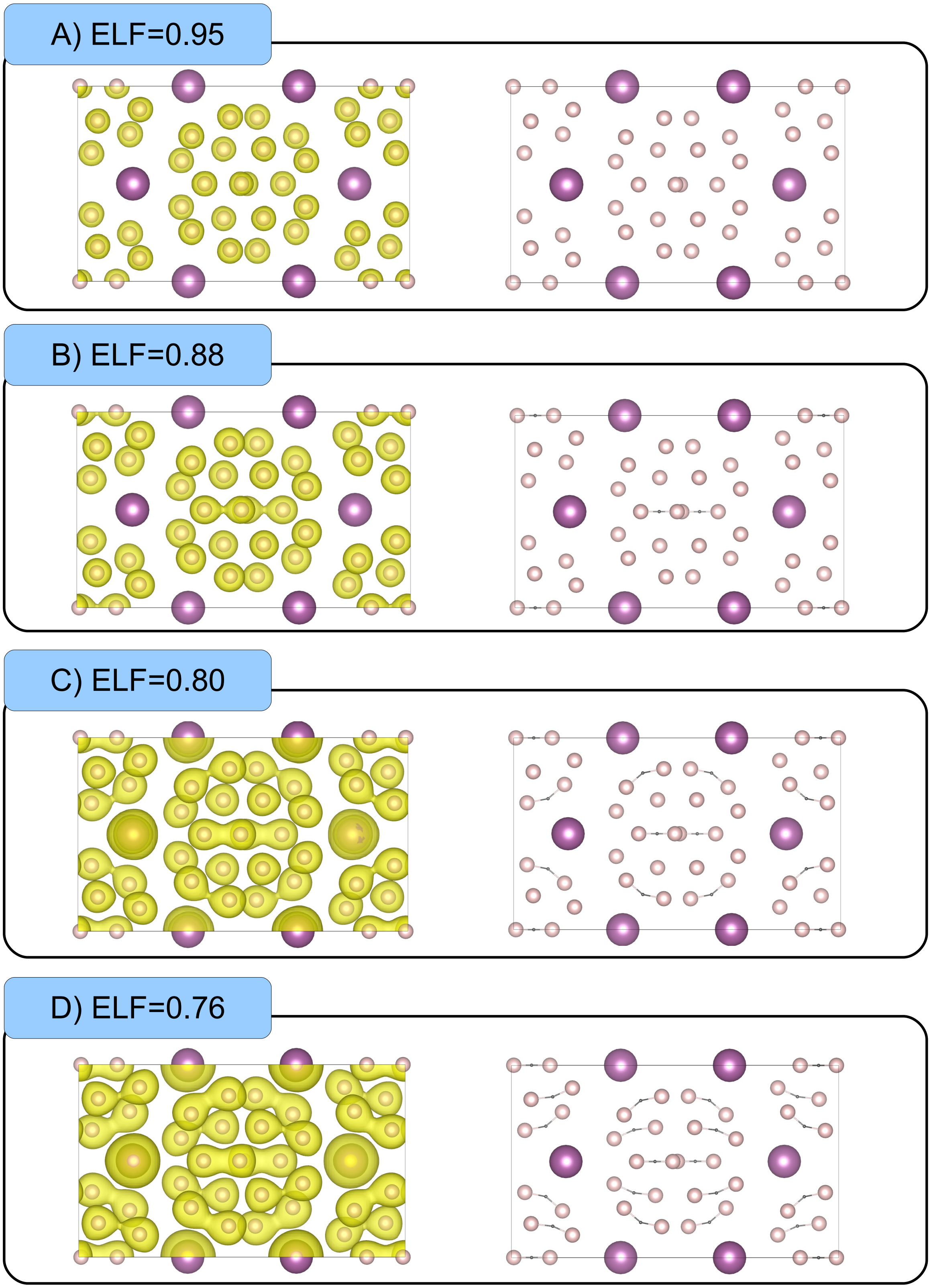
A) ELF=0.95
B) ELF=0.88
C) ELF=0.80
D) ELF=0.76

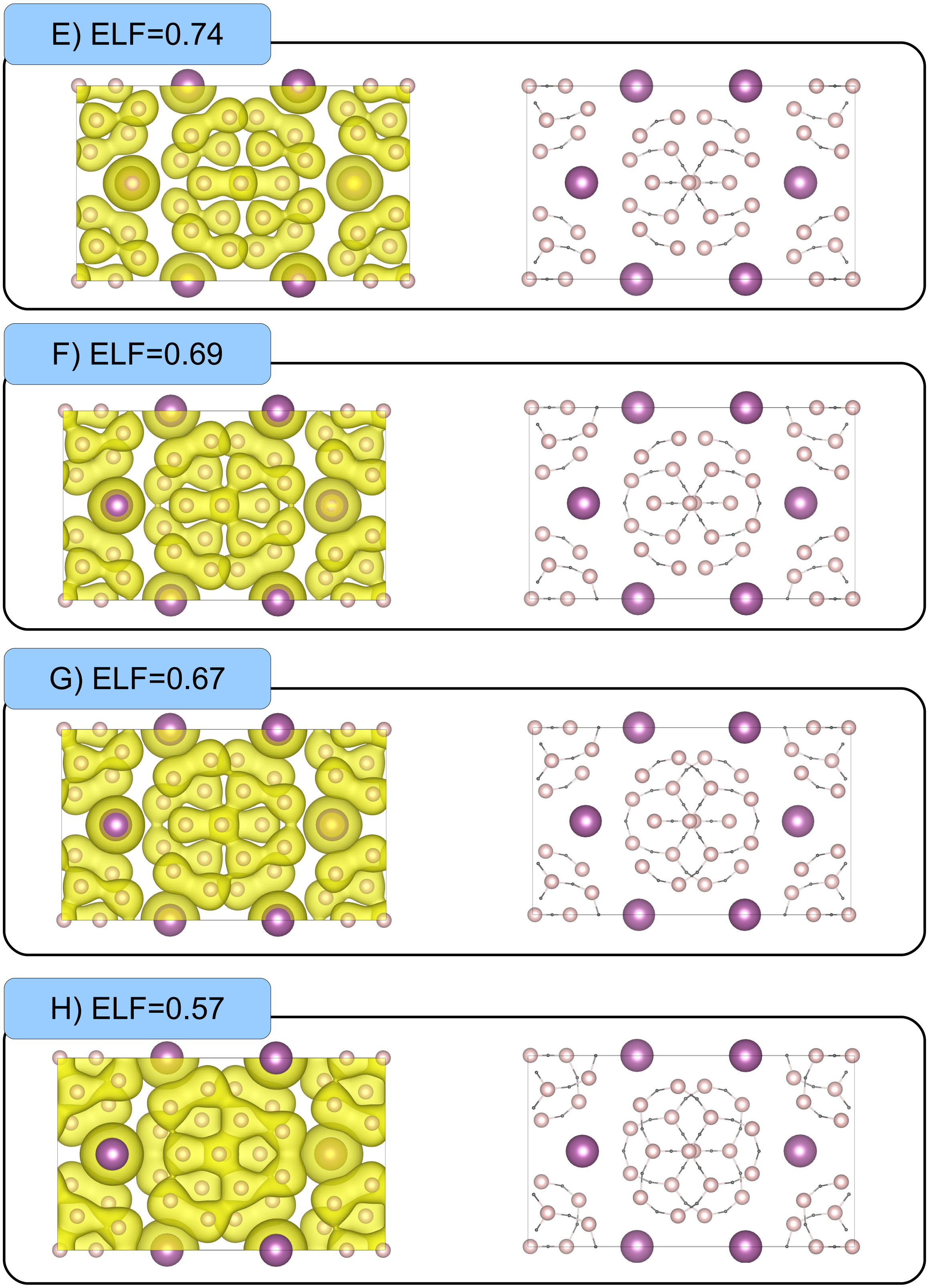
E) ELF=0.74
F) ELF=0.69
G) ELF=0.67
H) ELF=0.57

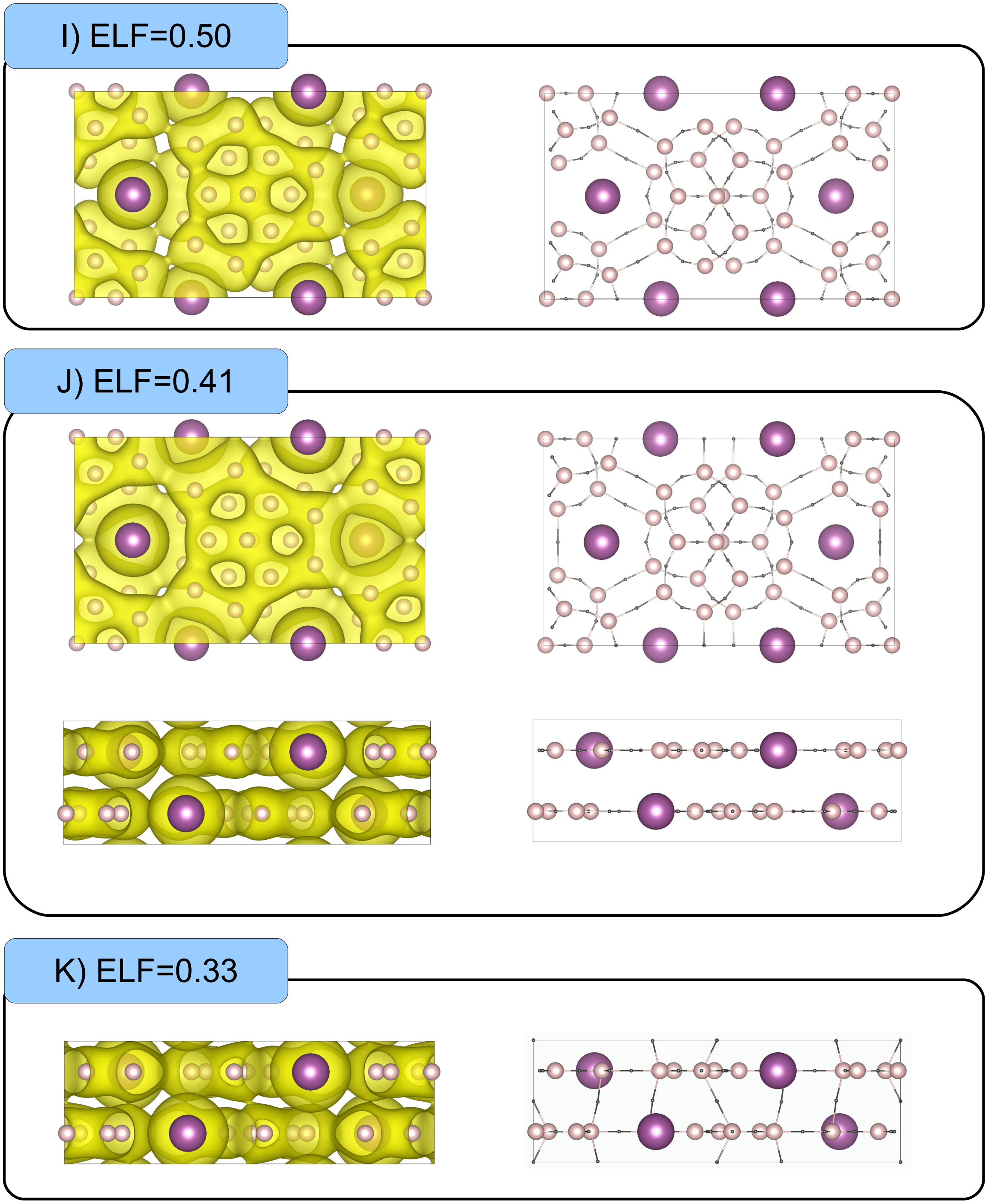

Figure 4. **Example of construction of Network 3.** The figure shows the steps for the construction of the network for ScH_{10} (32). On the left side are visualized the ELF isosurfaces, while on the right side are visualized the network patterns identified through the selection of the saddle points (small black dots) related to the same and higher value of ELF in respect to the ELF threshold in the blue box. From steps A) to J) is shown the progressive formation of the interatomic bonds leading to the creation of the network. In step J) can be seen that until for ELF$>$0.41 the formed network is 2D. In step K) the interplane ELF connection appears creating the 3D structure necessary for the determination of the *networking value* (ϕ). The resulting network includes all the hydrogen atoms.

DENSITY OF STATES

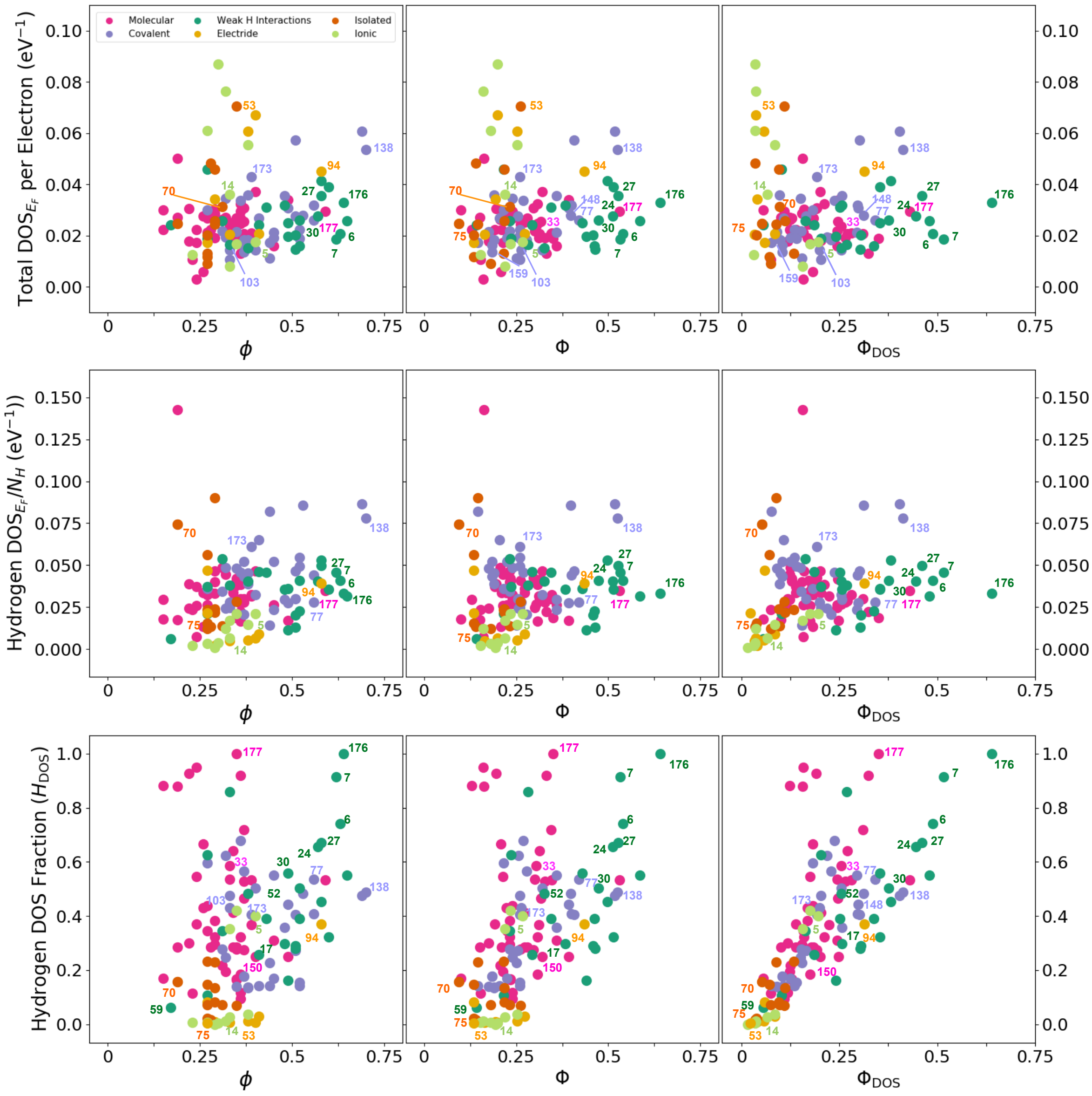

Figure 5. **DOS as a function of the** ***networking value*****.** The figure shows the total DOS per electron at the Fermi energy, the hydrogen contribution at the DOS at the Fermi energy per hydrogen atom, and the fraction of DOS at the Fermi level coming from the hydrogen orbitals as a function of the *networking value* (ϕ), the reduced networking value (Φ), and the reduced networking value weighted by the fraction of DOS at the Fermi level coming from the Hydrogen atoms (Φ_{DOS}) (See main text for the definitions).

SUPPLEMENTARY NOTE I: MODEL SUPERCONDUCTING ELECTRON LOCALIZATION FUNCTION

The *singlet pair function*, or Gor'kov function, for a BCS Cooper pair is given by:[1]

$$\Psi(\mathbf{r}_1 - \mathbf{r}_2) = \langle \Psi_{\mathrm{BCS}} \mid \psi_\downarrow(\mathbf{r}_2)\psi_\uparrow(\mathbf{r}_1) \mid \Psi_{\mathrm{BCS}} \rangle$$
$$= \frac{e^{i\theta}}{V} \sum_{\mathbf{k}} u_{\mathbf{k}} v_{\mathbf{k}} e^{-i\mathbf{k}\cdot(\mathbf{r}_1 - \mathbf{r}_2)},$$

where V is the volume, $v_{\mathbf{k}} = \sqrt{\frac{1}{2} - \frac{\varepsilon_{\mathbf{k}}}{2\sqrt{\varepsilon_{\mathbf{k}}^2 + \Delta_{\mathbf{k}}^2}}}$ and $u_{\mathbf{k}} = \sqrt{1 - v_{\mathbf{k}}^2}$, with $\varepsilon_{\mathbf{k}}$ the energies of an electron in a Fermi sea, and $\Delta_{\mathbf{k}}$ the superconducting gap. The singlet pair function represents the macroscopic probability amplitude of finding the electrons of the Cooper pair, with momentum $\mathbf{k}$ and $-\mathbf{k}$, at $\mathbf{r}_1$ and $\mathbf{r}_2$, respectively. In the case of radial $\mathbf{k}$, Kadin concluded that Ψ can be approximated by[2]

$$\Psi(r) \propto \cos(k_F r)\, K_0\left(\frac{r}{\pi \xi_0}\right),$$

where $\mathbf{r} = \mathbf{r}_1 - \mathbf{r}_2$, $\xi_0 = \frac{k_F}{\pi \Delta}$ is the BCS coherence length and, as in BCS, $\Delta_{\mathbf{k}} = \Delta$. In a local density approximation, $k_F = k_F(R) = (3\pi^2 \rho(R))^{1/3}$, we can define a Cooper pair wave function that also depends on its spatial position, R,

$$\Psi(r, R) \propto \cos(k_F(R) r)\, K_0\left(\frac{r}{\pi \xi_0}\right).$$

Taking the distance between the electrons equal to ξ_0,

$$\Psi(R) \equiv \Psi(\xi_0(R), R) \propto \cos\left(\frac{(9\pi\rho(R)^2)^{1/3}}{\Delta}\right) K_0\left(\frac{1}{\pi}\right). \tag{1}$$

which provides a real space representation of the Cooper pair wave function.

In Fig. 6 we show the ELF calculated both in the normal and superconducting state for a diatomic system in which two Gaussians centered at 0 and $2R_0$ represent the atomic cores and a diffuse function has been used to represent a metallic state. The results suggests that increasing the *networking value* also increases the delocalization (i.e. the connection between localized electrons) in the superconducting state, which can be assumed that helps superconductivity.

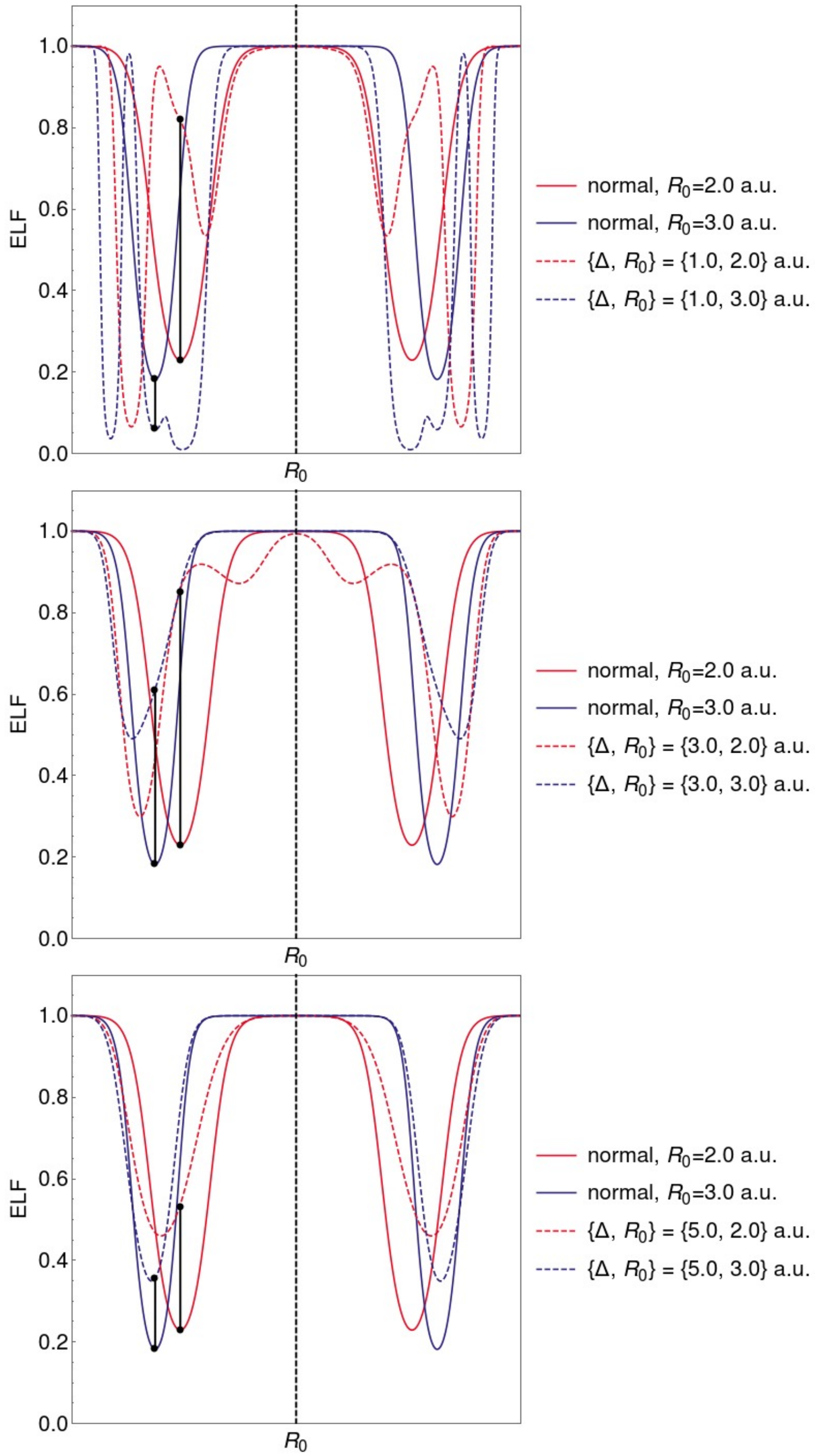

Figure 6. **ELF of the model superconducting wave function.** In the figures we show the value of the ELF calculated for the model of a diatomic system both in the normal state and in the superconducting state for different values of the superconducting gap and interatomic distance. The saddle point that determines the *networking value* has been marked with an arrow in each figure.

SUPPLEMENTARY TABLE

Table I: Table reporting item number used in the manuscript, chemical formula, space group, superconducting critical temperature as predicted, pressure at which it has has been predicted, hydrogen fraction H_f, and the reference from which the T_c value has been extracted. The empty cells refer to missing values from the literature or to the impossibility to perform calculations.

Item	Chemical formula	Space group	T_c (K)	Pressure (GPa)	H_f	Reference
0	LiH_2	$P4/mbm$	0	150	0.667	[3]
1	LiH_6	$R\bar{3}m$	38.34	150	0.857	[3]
2	LiH_8	$I422$	31.04	100	0.889	[3]
3	KH_6	$C2/c$	69.8	166	0.857	[4]
4	BeH_2	$Cmcm$	44.1	250	0.667	[5]
5	BeH_2	$P4/nmm$	62	400	0.667	[5]
6	MgH_6	$Im\bar{3}m$	271	400	0.857	[6]
7	CaH_6	$Im\bar{3}m$	235	150	0.857	[7]
8	SrH_6	$R\bar{3}m$	156	250	0.857	[8]
9	BaH_2	$R\bar{3}m$	0	60	0.667	[9]
10	BaH_6	$P4/mmm$	38	70	0.857	[9]
11	ScH_3	–	19.3	18	0.750	[10]
12	LaH_3	$Cmcm$	22.5	11	0.750	[10]
13	YH_3	–	40	17	0.750	[10]
14	ScH_2	–	32.9	80	0.667	[11]
15	YH_4	$I4/mmm$	95	120	0.800	[12]
16	YH_6	$Im\bar{3}m$	264	120	0.857	[12]
17	ScH_4	$I4/mmm$	98	200	0.800	[13]
18	ScH_6	$Im\bar{3}m$	169	350	0.857	[14]
19	PrH_9	$F\bar{4}3m$	0	100	0.900	[15]
20	CeH_{10}	$Fm\bar{3}m$	168	94	0.909	[16]
21	CeH_9	$P6_3/mmc$	50	100	0.900	[15]
22	LaH_9	Cc	30	50	0.900	[15]
23	LaH_6	$R\bar{3}c$	170	100	0.857	[15]
24	YH_9	$P6_3/mmc$	250	150	0.900	[15]
25	ScH_9	$P6_3/mmc$	180	400	0.900	[15]
27	YH_{10}	$Fm\bar{3}m$	326	250	0.909	[15, 17]
28	LaH_4	$I4/mmm$	10	300	0.800	[17]
29	LaH_8	$C2/m$	131	300	0.889	[17]
30	LaH_{10}	$Fm\bar{3}m$	274	150	0.909	[17]
31	ScH_9	$I4_1md$	163	300	0.900	[14]
32	ScH_{10}	$Cmcm$	120	250	0.909	[14]
33	ScH_{12}	$Immm$	141	350	0.923	[14]
34	ScH_7	$Cmcm$	169	300	0.875	[14]
35	ScH_3	$P6_3/mmc$	1	400	0.750	[14]
36	ScH_2	$P6/mmm$	4	300	0.667	[14]
37	ScH_6	$P6_3/mmc$	119	130	0.857	[8]
38	$TiD_{0.74}$	–	4.43	30	0.425	[18]
39	TiH_2	$Fm\bar{3}m$	7	0	0.667	[19]
40	TiH_2	$I4/mmm$	0	0	0.667	[19]
41	ZrH	$Cmcm$	11	120	0.500	[20]
42	HfH_2	$I4/mmm$	0	0	0.667	[21]
43	HfH_2	$Cmma$	8	180	0.667	[21]
44	HfH_2	$P2_1/m$	12	260	0.667	[21]
45	NbH_2	$P6_3mc$	0.5	60	0.667	[22]
46	VH_2	$Fm\bar{3}m$	0.5	0	0.667	[22]
47	NbH_2	$Fm\bar{3}m$	1.5	0	0.667	[22]
48	NbH_2	$Pnma$	4	60	0.667	[22]
49	NbH_4	$I4/mmm$	47	300	0.800	[23]
50	TaH_2	$Pnma$	7.1	200	0.667	[24]
51	TaH_4	$R\bar{3}m$	31	250	0.800	[24]
52	TaH_6	$Fdd2$	135.8	300	0.857	[24]
53	CrH	$P6_3/mmc$	10.6	0	0.500	[25]

54	CrH_3	$P6_3/mmc$	37.1	81	0.750	[25]
55	TcH_2	$I4/mmm$	10.64	200	0.667	[26]
56	TcH_2	$Cmcm$	8.61	300	0.667	[26]
57	TcH_3	$P4_2/mmc$	9.94	300	0.750	[26]
58	FeH_6	$Cmmm$	42.9	150	0.857	[27]
59	FeH_5	$I4/mmm$	45.8	150	0.833	[27]
60	FeH_5	$I4/mmm$	51	130	0.833	[28]
61	RuH	$Fm\bar{3}m$	0.41	100	0.500	[29]
62	RuH_3	$Pm\bar{3}m$	3.57	100	0.750	[29]
63	RuH_3	$Pm\bar{3}n$	1.25	200	0.750	[29]
64	OsH	$Fm\bar{3}m$	2.1	100	0.500	[30]
65	CoH	$Fm\bar{3}m$	0.11	5	0.500	[31]
66	RhH	$Fm\bar{3}m$	2.5	4	0.500	[8]
67	IrH	$Fm\bar{3}m$	7	80	0.500	[8]
68	PdH	–	9	0	0.500	[32]
69	PdD	–	11	0	0.500	[32]
70	PdH	$Fm\bar{3}m$	5	0	0.500	[33]
71	PdD	$Fm\bar{3}m$	6.5	0	0.500	[33]
72	PdT	$Fm\bar{3}m$	6.9	0	0.500	[33]
73	PtH	$Fm\bar{3}m$	15	100	0.500	[33]
74	PtH	$P6_3/mmc$	25	80	0.500	[33]
75	AuH	$Fm\bar{3}m$	21	220	0.500	[8]
76	BH	$P6/mmm$	21.4	175	0.500	[34]
77	BH_3	$Pbcn$	125	360	0.750	[35]
78	AlH_3	$Pm\bar{3}n$	11.5	73	0.750	[36, 37]
79	$AlH_3(H_2)$	$P2_1/m$	146	250	0.750	[38]
80	GaH_3	$Pm\bar{3}n$	102	120	0.750	[39]
81	InH_5	$P2_1/m$	27.1	150	0.833	[40]
82	InH_3	$R\bar{3}$	40.5	200	0.750	[40]
83	SiH_4	$C2/c$	55	125	0.800	[41]
84	SiH_4	$P6/mmm$	74	120	0.800	[42]
85	SiH_4	$Pmna$	166	202	0.800	[43]
86	SiH_4	$C2/c$	30	300	0.800	[44]
87	SiH_4	$P2_1/c$	35	400	0.800	[44]
88	SiH_4	$C2/m$	110	610	0.800	[44]
89	SiH_4	$P\bar{3}$	35.1	300	0.800	[44]
90	SiH_4	$Cmca$	20	150	0.800	[45]
91	SiH_4	$Pbcn$	16.5	190	0.800	[46]
92	SiH_4H_2	$Cmca$	107	250	0.857	[47]
93	Si_2H_6	$P\bar{1}$	80	200	0.750	[48]
94	Si_2H_6	$Pm\bar{3}m$	153	275	0.750	[48]
95	Si_2H_6	$C2/c$	42	300	0.750	[48]
96	Si_2H_6	$Cmcm$	25	100	0.750	[49]
97	GeH_4	$C2/c$	64	220	0.800	[50]
98	GeH_4	$Cmmm$	47	20	0.800	[51]
99	GeH_4	$Ama2$	57	250	0.800	[52]
100	GeH_4	$C2/c$	84	500	0.800	[52]
101	$GeH_4(H_2)2$	$P2_1/c$	90	250	0.889	[53]
102	GeH_3	$Cccm$	80	300	0.857	[54]
103	GeH_4	$C2/m$	67	280	0.800	[55]
104	$Ge3H_{11}$	$I\bar{4}m2$	43	285	0.786	[55]
105	GeH_3	$P4_2/mmc$	90	180	0.750	[56]
106	GeH_3	$Pm\bar{3}m$	140	180	0.750	[56]
107	SnH_8	$I\bar{4}m2$	72	250	0.889	[57]
108	SnH_4	$Ama2$	22	120	0.800	[58]
109	SnH_4	$P6_3/mmc$	62	200	0.800	[58]
110	SnH_4	$P6/mmm$	80	120	0.800	[59]
111	SnH_4	$C2/m$	95	600	0.800	[60]
112	SnH_4	$I4/mmm$	91	220	0.800	[61]
113	SnH_{12}	$C2/m$	93	250	0.923	[61]
114	SnH_{14}	$C2/m$	97	300	0.933	[61]
115	$PbH_4(H_2)2$	$C2/m$	107	230	0.889	[61]
116	PH_3	0	100	226	0.750	[62]
117	PH	$I4/mmm$	81	250	0.500	[63]

118	PH_2	$I4/mmm$	86	260	0.667	[63, 64]
119	PH_4	$C2/m$	1.9	80	0.800	[65]
120	PH_2	$Cmmm$	29.5	80	0.667	[65]
121	AsH	$Cmcm$	21.2	300	0.500	[66]
122	AsH_8	$C2/c$	151.4	450	0.889	[66]
123	SbH	$Pnma$	14.6	175	0.500	[66]
124	SbH_3	$Pmmn$	25.9	300	0.750	[66]
125	SbH_4	$P6_3/mmc$	102.2	150	0.800	[66]
126	BiH_2	$Pnma$	39	125	0.667	[67]
127	BiH_3	$I4_1/amd$	65	270	0.750	[67]
128	SbH_3	$Pnma$	68	170	0.750	[67]
129	BiH	$P6_3/mmc$	30	250	0.500	[68]
130	BiH_2	$P2_1/m$	65	300	0.667	[68]
131	BiH_4	$Pmmm$	93	150	0.800	[68]
132	BiH_5	$C2/m$	119	300	0.833	[68]
133	BiH_6	$P\bar{1}$	113	300	0.857	[68]
134	H_2S	$Cmca$	82	160	0.500	[69]
135	H_2S	$P\bar{1}$	60	158	0.500	[69]
136	$(H_2S)2H_2$	$Im\bar{3}m$	204	200	0.750	[70]
137	$(H_2S)2H_2$	$R\bar{3}m$	166	130	0.750	[70]
138	H_3S	$Im\bar{3}m$	225	150	0.750	[71]
139	H_3S	$R\bar{3}m$	214	170	0.750	[72]
140	H_4S_3	$Pnma$	2.1	140	0.571	[73]
141	H_5S_2	$P\bar{1}$	79	130	0.714	[74]
142	D_3S	$Im\bar{3}m$	188	200	0.750	[75]
143	$H_3S_{0.925}P_{0.075}$	$Im\bar{3}m$	280	250	0.750	[76]
144	$H_3S_{0.9}P_{0.1}$	$Im\bar{3}m$	240	200	0.750	[76]
145	$H_3S_{0.96}Si_{0.04}$	$Im\bar{3}m$	275	250	0.750	[76]
146	HSe_2	$C2/m$	5	300	0.333	[77]
147	HSe	$P4/nmm$	42	300	0.500	[77]
148	H_3Se	$Im\bar{3}m$	116	200	0.750	[77]
149	HSe	$P2_1/c$	23	300	0.500	[77]
150	H_4Te	$P6/mmm$	104	170	0.800	[78]
151	H_5Te_2	$C2/m$	58	200	0.714	[78]
152	HTe	$P4/nmm$	28	150	0.500	[78]
153	H_4Te	$R\bar{3}m$	76	270	0.800	[78]
154	HTe	$P6_3/mmc$	44.2	300	0.500	[78]
155	PoH_4	$C2/c$	53.6	250	0.800	[79]
156	PoH	$P6_3/mmc$	0.65	300	0.500	[79]
157	PoH_2	$Pnma$	0	200	0.667	[79]
158	PoH_6	$C2/m$	4.68	200	0.857	[79]
159	HBr	$P2_1/m$	51	200	0.500	[80]
160	HCl	$P2_1/m$	40	360	0.500	[80]
161	HBr	$C2/m$	27	150	0.500	[81]
162	HCl	$C2/m$	20	250	0.500	[82]
163	H_2I	$Cmcm$	8	100	0.667	[83]
164	H_4I	$P6/mmm$	12.5	300	0.800	[84]
165	H_2I	$Pnma$	5.3	100	0.667	[84]
166	H_2I	$R\bar{3}m$	33	240	0.667	[84]
167	XeH	$Immm$	28	100	0.500	[85]
168	XeH_2	$Cmcm$	26	400	0.667	[85]
169	MgH_2	$P6_3/mmc$	23	180	0.667	[86]
170	MgH_4	$Cmcm$	37	100	0.800	[86]
171	MgH_{12}	$R\bar{3}$	60	140	0.923	[86]
172	PH_3	$C2/m$	71	260	0.750	[87, 88]
173	PH_2	$C2/m$	95	260	0.667	[87, 88]
174	H_2Br	$Cmcm$	12.1	240	0.667	[89]
175	H_4Br	$P6_3/mmc$	2.4	240	0.800	[89]
176	H	$I4_1/amd$	318	500	1.000	[100]
177	H	$Cmca\bar{4}$	109	450	1.000	[90]

[1] J. R. Waldran. *Superconductivity of metals and cuprates.* Institute of Physics, 1996.
[2] A. Kadin. *J. Supercond. Nov. Magn.*, **20**, 285–292, (2007).
[3] Yu Xie, Quan Li, Artem R. Oganov, and Hui Wang. Superconductivity of lithium-doped hydrogen under high pressure. *Acta Crystallographica Section C*, **70**, 104-111, (2014).
[4] Dawei Zhou, Xilian Jin, Xing Meng, Gang Bao, Yanming Ma, Bingbing Liu, and Tian Cui. Ab initio study revealing a layered structure in hydrogen-rich KH6 under high pressure. *Phys. Rev. B*, **86**, 014118, (2012).
[5] Shuyin Yu, Qingfeng Zeng, Artem R. Oganov, Chaohao Hu, Gilles Frapper, and Litong Zhang. Exploration of stable compounds, crystal structures, and superconductivity in the Be-H system. *AIP Advances*, **4**, 107118, (2014).
[6] Xiaolei Feng, Jurong Zhang, Guoying Gao, Hanyu Liu, and Hui Wang. Compressed sodalite-like mgh6 as a potential high-temperature superconductor. *RSC Adv.*, **5**, 59292-59296, (2015).
[7] Hui Wang, John S. Tse, Kaori Tanaka, Toshiaki Iitaka, and Yanming Ma. Superconductive sodalite-like clathrate calcium hydride at high pressures. *Proceedings of the National Academy of Sciences*, (109), 6463-6466, (2012).
[8] Tiange Bi, Niloofar Zarifi, Tyson Terpstra, and Eva Zurek. The search for superconductivity in high pressure hydrides. *Reference Module in Chemistry, Molecular Sciences and Chemical Engineering.* Elsevier, (2019).
[9] James Hooper, Bahadir Altintas, Andrew Shamp, and Eva Zurek. Polyhydrides of the alkaline earth metals: A look at the extremes under pressure. *The Journal of Physical Chemistry C*, **117**, 2982-2992, (2013).
[10] Duck Young Kim, Ralph H. Scheicher, Ho-kwang Mao, Tae W. Kang, and Rajeev Ahuja. General trend for pressurized superconducting hydrogen-dense materials. *Proceedings of the National Academy of Sciences*, **107**, 2793-2796, (2010).
[11] Yong-Kai Wei, Jiao-Nan Yuan, Faez Iqbal Khan, Guang-Fu Ji, Zhuo-Wei Gu, and Dong-Qing Wei. Pressure induced superconductivity and electronic structure properties of scandium hydrides using first principles calculations. *RSC Adv.*, **6**, 81534-81541, (2016).
[12] Yinwei Li, Jian Hao, Hanyu Liu, S Tse John, Yanchao Wang, and Yanming Ma. Pressure-stabilized superconductive yttrium hydrides. *Scientific Reports*, **5**, 9948, (2015).
[13] Shifeng Qian, Xiaowei Sheng, Xiaozhen Yan, Yangmei Chen, and Bo Song. Theoretical study of stability and superconductivity of ScH_n $(n = 4 - 8)$ at high pressure. *Phys. Rev. B*, **96**, 094513, (2017).
[14] Xiaoqiu Ye, Niloofar Zarifi, Eva Zurek, Roald Hoffmann, and NW Ashcroft. High hydrides of scandium under pressure: potential superconductors. *J. Phys. Chem. C*, **122**, 6298-6309, (2018).
[15] Feng Peng, Ying Sun, Chris J. Pickard, Richard J. Needs, Qiang Wu, and Yanming Ma. Hydrogen clathrate structures in rare earth hydrides at high pressures: Possible route to room-temperature superconductivity. *Phys. Rev. Lett.*, **119**, 107001, (2017).
[16] Bin Li, Zilong Miao, Lei Ti, Shengli Liu, Jie Chen, Zhixiang Shi, and Eugene Gregoryanz. Predicted high-temperature superconductivity in cerium hydrides at high pressures. *Journal of Applied Physics*, **126**, 235901, (2019).
[17] Hanyu Liu, Ivan I. Naumov, Roald Hoffmann, N. W. Ashcroft, and Russell J. Hemley. Potential high-Tc superconducting lanthanum and yttrium hydrides at high pressure. *Proceedings of the National Academy of Sciences*, **114**, 6990-6995, (2017).
[18] IO Bashkin, MV Nefedova, VG Tissen, and EG Ponyatovskii. Superconductivity in the Ti-d system under pressure. *Physics of the Solid State*, **40**, 1950-1952, (1998).
[19] Kavungal Veedu Shanavas, L Lindsay, and David S Parker. Electronic structure and electron-phonon coupling in TiH 2. *Scientific Reports*, **6**, 28102, (2016).
[20] Xiao-Feng Li, Zi-Yu Hu, and Bing Huang. Phase diagram and superconductivity of compressed zirconium hydrides. *Phys. Chem. Chem. Phys.*, **19**, 3538-3543, (2017).
[21] Yunxian Liu, Xiaoli Huang, Defang Duan, Fubo Tian, Hanyu Liu, Da Li, Zhonglong Zhao, Xiaojing Sha, Hongyu Yu, Huadi Zhang, et al. First-principles study on the structural and electronic properties of metallic HfH_2 under pressure. *Scientific Reports*, **5**, 11381, (2015).
[22] Changbo Chen, Fubo Tian, Defang Duan, Kuo Bao, Xilian Jin, Bingbing Liu, and Tian Cui. Pressure induced phase transition in MH_2 (M = V, Nb). *J. Chem. Phys*, **140**, 114703, (2014).
[23] Guoying Gao, Roald Hoffmann, N. W. Ashcroft, Hanyu Liu, Aitor Bergara, and Yanming Ma. Theoretical study of the ground-state structures and properties of niobium hydrides under pressure. *Phys. Rev. B*, **88**, 184104, (2013).
[24] Quan Zhuang, Xilian Jin, Tian Cui, Yanbin Ma, Qianqian Lv, Ying Li, Huadi Zhang, Xing Meng, and Kuo Bao. Pressure-stabilized superconductive ionic tantalum hydrides. *Inorganic chemistry*, **56**, 3901-3908, (2017).
[25] Shuyin Yu, Xiaojing Jia, Gilles Frapper, Duan Li, Artem R Oganov, Qingfeng Zeng, and Litong Zhang. Pressure-driven formation and stabilization of superconductive chromium hydrides. *Scientific Reports*, **5**, 17764, (2015).
[26] Xiaofeng Li, Hanyu Liu, and Feng Peng. Crystal structures and superconductivity of technetium hydrides under pressure. *Phys. Chem. Chem. Phys.*, **18**, 28791-28796, (2016).
[27] Alexander G Kvashnin, Ivan A Kruglov, Dmitrii V Semenok, and Artem R Oganov. Iron superhydrides FeH_5 and FeH_6: stability, electronic properties, and superconductivity. *J. Phys. Chem. C*, **122**, 4731-4736, (2018).
[28] Arnab Majumdar, John S. Tse, Min Wu, and Yansun Yao. Superconductivity in FeH_5. *Phys. Rev. B*, **96**, 201107, (2017).
[29] Yunxian Liu, Defang Duan, Fubo Tian, Chao Wang, Yanbin Ma, Da Li, Xiaoli Huang, Bingbing Liu, and Tian Cui. Stability and properties of the Ru-H system at high pressure. *Phys. Chem. Chem. Phys.*, **18**, 1516-1520, (2016).
[30] Yunxian Liu, Defang Duan, Xiaoli Huang, Fubo Tian, Da Li, Xiaojing Sha, Chao Wang, Huadi Zhang, Ting Yang, Bingbing Liu, et al. Structures and properties of osmium hydrides under pressure from first principle calculation. *J. Phys.*

Chem. C, **119**, 15905-15911, (2015).
[31] Liyuan Wang, Defang Duan, Hongyu Yu, Hui Xie, Xiaoli Huang, Yanbin Ma, Fubo Tian, Da Li, Bingbing Liu, and Tian Cui. High-pressure formation of cobalt polyhydrides: A first-principle study. *Inorganic Chemistry*, **57**, 181-186, (2018).
[32] B Stritzker and W Buckel. Superconductivity in the palladium-hydrogen and the palladium-deuterium systems. *Zeitschrift für Physik A Hadrons and nuclei*, **257**, 1-8, (1972).
[33] Ion Errea, Matteo Calandra, and Francesco Mauri. Anharmonic free energies and phonon dispersions from the stochastic self-consistent harmonic approximation: Application to platinum and palladium hydrides. *Phys. Rev. B*, **89**, 064302, (2014).
[34] Chao-Hao Hu, Artem R Oganov, Qiang Zhu, Guang-Rui Qian, Gilles Frapper, Andriy O Lyakhov, and Huai-Ying Zhou. Pressure-induced stabilization and insulator-superconductor transition of BH. *Phys. Rev. Lett.*, **110**, 165504, (2013).
[35] Kazutaka Abe and NW Ashcroft. Crystalline diborane at high pressures. *Phys. Rev. B*, **84**, 104118, (2011).
[36] Yong-Kai Wei, Ni-Na Ge, Guang-Fu Ji, Xiang-Rong Chen, Ling-Cang Cai, Su-Qin Zhou, and Dong-Qing Wei. Elastic, superconducting, and thermodynamic properties of the cubic metallic phase of AlH_3 via first-principles calculations. *Journal of Applied Physics*, **114**, 114905, (2013).
[37] Igor Goncharenko, MI Eremets, M Hanfland, JS Tse, M Amboage, Y Yao, and IA Trojan. Pressure-induced hydrogen-dominant metallic state in aluminum hydride. *Phys. Rev. Lett.*, **100**, 045504, (2008).
[38] Pugeng Hou, Xiusong Zhao, Fubo Tian, Da Li, Defang Duan, Zhonglong Zhao, Binhua Chu, Bingbing Liu, and Tian Cui. High pressure structures and superconductivity of $AlH_3(H_2)$ predicted by first principles. *RSC Adv.*, **5**, 5096-5101, (2015).
[39] Guoying Gao, Hui Wang, Aitor Bergara, Yinwei Li, Guangtao Liu, and Yanming Ma. Metallic and superconducting gallane under high pressure. *Phys. Rev. B*, **84**, 064118, (2011).
[40] Yunxian Liu, Defang Duan, Fubo Tian, Hanyu Liu, Chao Wang, Xiaoli Huang, Da Li, Yanbin Ma, Bingbing Liu, and Tian Cui. Pressure-induced structures and properties in indium hydrides. *Inorganic chemistry*, **54**, 9924-9928, (2015).
[41] Y Yao, J. S Tse, Y Ma, and K Tanaka. Superconductivity in high-pressure SiH4. *Europhysics Letters*, **78**, 37003, (2007).
[42] JS Tse, Y Yao, and K Tanaka. Novel superconductivity in metallic SnH_4 under high pressure. *Phys. Rev. Lett.*, **98**, 117004, (2007).
[43] Ji Feng, Wojciech Grochala, Tomasz Jaroń, Roald Hoffmann, Aitor Bergara, and NW Ashcroft. Structures and potential superconductivity in SiH_4 at high pressure: En route to "metallic hydrogen". *Phys. Rev. Lett.*, **96**, 017006, (2006).
[44] Huadi Zhang, Xilian Jin, Yunzhou Lv, Quan Zhuang, Yunxian Liu, Qianqian Lv, Kuo Bao, Da Li, Bingbing Liu, and Tian Cui. High-temperature superconductivity in compressed solid silane. *Scientific Reports*, **5**, 8845, (2015).
[45] Xiao-Jia Chen, Jiang-Long Wang, Viktor V Struzhkin, Ho-kwang Mao, Russell J Hemley, and Hai-Qing Lin. Superconducting behavior in compressed solid SiH_4 with a layered structure. *Phys. Rev. Lett.*, **101**, 077002, (2008).
[46] Miguel Martinez-Canales, Artem R Oganov, Yanming Ma, Yan Yan, Andriy O Lyakhov, and Aitor Bergara. Novel structures and superconductivity of silane under pressure. *Phys. Rev. Lett.*, **102**, 087005, (2009).
[47] Yinwei Li, Guoying Gao, Yu Xie, Yanming Ma, Tian Cui, and Guangtian Zou. Superconductivity at 100 K in dense $SiH_4(H_2)2$ predicted by first principles. *Proceedings of the National Academy of Sciences*, **107**, 15708-15711, (2010).
[48] Xilian Jin, Xing Meng, Zhi He, Yanming Ma, Bingbing Liu, Tian Cui, Guangtian Zou, and Ho-kwang Mao. Superconducting high-pressure phases of disilane. *Proceedings of the National Academy of Sciences*, **107**, 9969-9973, (2010).
[49] Jos'e A. Flores-Livas, Maximilian Amsler, Thomas J. Lenosky, Lauri Lehtovaara, Silvana Botti, Miguel A. L. Marques, and Stefan Goedecke High-pressure structures of disilane and their superconducting properties *Phys. Rev. Lett.*, **108**, 117004, (2012).
[50] Guoying Gao, Artem R Oganov, Aitor Bergara, Miguel Martinez-Canales, Tian Cui, Toshiaki Iitaka, Yanming Ma, and Guangtian Zou. Superconducting high pressure phase of germane. *Phys Rev. Lett.*, **101**, 107002, (2008).
[51] Chao Zhang, Xiao-Jia Chen, Yan-Ling Li, Viktor V Struzhkin, Russell J Hemley, Ho-Kwang Mao, Rui-Qin Zhang, and Hai-Qing Lin. Superconductivity in hydrogen-rich material: GeH_4. *Journal of superconductivity and novel magnetism*, **23**, 717-719, (2010).
[52] Huadi Zhang, Xilian Jin, Yunzhou Lv, Quan Zhuang, Qianqian Lv, Yunxian Liu, Kuo Bao, Da Li, Bingbing Liu, and Tian Cui. Investigation of stable germane structures under high-pressure. *Phys. Chem. Chem. Phys.*, **17**, 27630-27635, (2015).
[53] Guohua Zhong, Chao Zhang, Xiaojia Chen, Yanling Li, Ruiqin Zhang, and Haiqing Lin. Structural, electronic, dynamical, and superconducting properties in dense $GeH_4(H_2)2$. *J. Phys. Chem. C*, **116**, 5225-5234, (2012).
[54] PuGeng Hou, FuBo Tian, Da Li, ZhongLong Zhao, DeFang Duan, HuaDi Zhang, XiaoJing Sha, BingBing Liu, and Tian Cui. Ab initio study of germanium-hydride compounds under high pressure. *RSC Adv.*, **5**, 19432-19438, (2015).
[55] M Mahdi Davari Esfahani, Artem R Oganov, Haiyang Niu, and Jin Zhang. Superconductivity and unexpected chemistry of germanium hydrides under pressure. *Phys. Rev. B*, **95**, 134506, (2017).
[56] Kazutaka Abe and NW Ashcroft. Quantum disproportionation: The high hydrides at elevated pressures. *Phys. Rev. B*, **88**, 174110, (2013).
[57] Huadi Zhang, Xilian Jin, Yunzhou Lv, Quan Zhuang, Yunxian Liu, Qianqian Lv, Da Li, Kuo Bao, Bingbing Liu, and Tian Cui. A novel stable hydrogen-rich SnH_8 under high pressure. *RSC Adv.*, **5**, 107637-107641, (2015).
[58] Guoying Gao, Artem R Oganov, Peifang Li, Zhenwei Li, Hui Wang, Tian Cui, Yanming Ma, Aitor Bergara, Andriy O Lyakhov, Toshiaki Iitaka, et al. High-pressure crystal structures and superconductivity of stannane (SnH_4). *Proceedings of the National Academy of Sciences*, **107**, 1317-1320, (2010).
[59] JS Tse, Y Yao, and K Tanaka. Novel superconductivity in metallic snh 4 under high pressure. *Phys. Rev. Let.*, **98**, 117004, (2007).
[60] Huadi Zhang, Xilian Jin, Yunzhou Lv, Quan Zhuang, Ying Li, Kuo Bao, Da Li, Bingbing Liu, and Tian Cui. Pressure-induced phase transition of SnH_4: a new layered structure. *RSC Adv.*, **6**, 10456-10461, (2016).

[61] M Mahdi Davari Esfahani, Zhenhai Wang, Artem R Oganov, Huafeng Dong, Qiang Zhu, Shengnan Wang, Maksim S Rakitin, and Xiang-Feng Zhou. Superconductivity of novel tin hydrides (Sn_nH_m) under pressure. *Scientific Reports*, **6**, 22873, (2016).
[62] I. A. Troyan A.P. Drozdov, M. I. Eremets. Superconductivity above 100 K in PH_3 at high pressures. *ArXiv preprint arXiv:1508.06224*, (2015).
[63] José A Flores-Livas, Maximilian Amsler, Christoph Heil, Antonio Sanna, Lilia Boeri, Gianni Profeta, Chris Wolverton, Stefan Goedecker, and EKU Gross. Superconductivity in metastable phases of phosphorus-hydride compounds under high pressure. *Phys. Rev. B*, **93**, 020508, (2016).
[64] Hanyu Liu, Yinwei Li, Guoying Gao, John S Tse, and Ivan I Naumov. Crystal structure and superconductivity of ph3 at high pressures. *J. Phys. Chem. C*, **120**, 3458-3461, (2016).
[65] Tiange Bi, Daniel P Miller, Andrew Shamp, and Eva Zurek. Superconducting phases of phosphorus hydride under pressure: stabilization by mobile molecular hydrogen. *Angewandte Chemie*, **129**, 10326-10329, (2017).
[66] Yuhao Fu, Xiangpo Du, Lijun Zhang, Feng Peng, Miao Zhang, Chris J Pickard, Richard J Needs, David J Singh, Weitao Zheng, and Yanming Ma. High-pressure phase stability and superconductivity of pnictogen hydrides and chemical trends for compressed hydrides. *Chemistry of Materials*, **28**, 1746-1755, (2016).
[67] Kazutaka Abe and NW Ashcroft. Stabilization and highly metallic properties of heavy group-v hydrides at high pressures. *Phys. Rev. B*, **92**, 224109, (2015).
[68] Yanbin Ma, Defang Duan, Da Li, Yunxian Liu, Fubo Tian, Hongyu Yu, Chunhong Xu, Ziji Shao, Bingbing Liu, and Tian Cui. High-pressure structures and superconductivity of bismuth hydrides. *arXiv preprint arXiv:1511.05291*, (2015).
[69] Yinwei Li, Jian Hao, Hanyu Liu, Yanling Li, and Yanming Ma. The metallization and superconductivity of dense hydrogen sulfide. *J. Chem. Phys.*, **140**, 174712, (2014).
[70] Defang Duan, Yunxian Liu, Fubo Tian, Da Li, Xiaoli Huang, Zhonglong Zhao, Hongyu Yu, Bingbing Liu, Wenjing Tian, and Tian Cui. Pressure-induced metallization of dense $(H_2S)2H_2$ with high-T_c superconductivity. *Scientific Reports*, **4**, 6968, (2014).
[71] Ion Errea, Matteo Calandra, Chris J. Pickard, Joseph R. Nelson, Richard J. Needs, Yinwei Li, Hanyu Liu, Yunwei Zhang, Yanming Ma, and Francesco Mauri. Quantum hydrogen-bond symmetrization in the superconducting hydrogen sulfide system. *Nature*, **532**, 81-84, (2016).
[72] Ryosuke Akashi, Mitsuaki Kawamura, Shinji Tsuneyuki, Yusuke Nomura, and Ryotaro Arita. First-principles study of the pressure and crystal-structure dependences of the superconducting transition temperature in compressed sulfur hydrides. *Phys. Rev. B*, **91**, 224513, (2015).
[73] Yinwei Li, Lin Wang, Hanyu Liu, Yunwei Zhang, Jian Hao, Chris J Pickard, Joseph R Nelson, Richard J Needs, Wentao Li, Yanwei Huang, et al. Dissociation products and structures of solid H_2S at strong compression. *Phys. Rev. B*, **93**, 020103, (2016).
[74] Takahiro Ishikawa, Akitaka Nakanishi, Katsuya Shimizu, Hiroshi Katayama-Yoshida, Tatsuki Oda, and Naoshi Suzuki. Superconducting H_5S_2 phase in sulfur-hydrogen system under high-pressure. *Scientific Reports*, **6**, 23160, (2016).
[75] José A Flores-Livas, Antonio Sanna, and EKU Gross. High temperature superconductivity in sulfur and selenium hydrides at high pressure. *The European Physical Journal B*, **89**, 63, (2016).
[76] Yanfeng Ge, Fan Zhang, and Yugui Yao. First-principles demonstration of superconductivity at 280 K in hydrogen sulfide with low phosphorus substitution. *Phys. Rev. B*, **93**, 224513, (2016).
[77] Shoutao Zhang, Yanchao Wang, Jurong Zhang, Hanyu Liu, Xin Zhong, Hai-Feng Song, Guochun Yang, Lijun Zhang, and Yanming Ma. Phase diagram and high-temperature superconductivity of compressed selenium hydrides. *Scientific Reports*, **5**, 1-8, (2015).
[78] Xin Zhong, Hui Wang, Jurong Zhang, Hanyu Liu, Shoutao Zhang, Hai-Feng Song, Guochun Yang, Lijun Zhang, and Yanming Ma. Tellurium hydrides at high pressures: High-temperature superconductors. *Phys. Rev. Lett.*, **116**, 057002, (2016).
[79] Yunxian Liu, Defang Duan, Fubo Tian, Chao Wang, Gang Wu, Yanbin Ma, Hongyu Yu, Da Li, Bingbing Liu, and Tian Cui. Prediction of stoichiometric poh n compounds: crystal structures and properties. *RSC Adv.*, **5**, 103445-103450, (2015).
[80] Defang Duan, Fubo Tian, Zhi He, Xing Meng, Liancheng Wang, Changbo Chen, Xiusong Zhao, Bingbing Liu, and Tian Cui. Hydrogen bond symmetrization and superconducting phase of HBr and HCl under high pressure: An ab initio study. *J. Chem. Phys.*, **133**, 074509, (2010).
[81] Siyu Lu, Min Wu, Hanyu Liu, S Tse John, and Bai Yang. Prediction of novel crystal structures and superconductivity of compressed HBr. *RSC Adv.*, **5**, 45812-45816, (2015).
[82] Changbo Chen, Ying Xu, Xiuping Sun, and Sihan Wang. Novel superconducting phases of hcl and hbr under high pressure: an ab initio study. *J.Phys. Chem. C*, **119**, 17039-17043, (2015).
[83] Andrew Shamp and Eva Zurek. Superconducting high-pressure phases composed of hydrogen and iodine. *J. Phys. Chem. Lett.*, **6**, 4067-4072, (2015).
[84] Defang Duan, Fubo Tian, Yunxian Liu, Xiaoli Huang, Da Li, Hongyu Yu, Yanbin Ma, Bingbing Liu, and Tian Cui. Enhancement of T_c in the atomic phase of iodine-doped hydrogen at high pressures. *Phys. Chem. Chem. Phys.*, **17**, 32335-32340, (2015).
[85] Xiaozhen Yan, Yangmei Chen, Xiaoyu Kuang, and Shikai Xiang. Structure, stability, and superconductivity of new Xe-H compounds under high pressure. *J. Chem. Phys.*, **143**, 124310, (2015).
[86] David C Lonie, James Hooper, Bahadir Altintas, and Eva Zurek. Metallization of magnesium polyhydrides under pressure. *Phys. Rev. B*, **87**, 054107, (2013).
[87] José A Flores-Livas, Maximilian Amsler, Christoph Heil, Antonio Sanna, Lilia Boeri, Gianni Profeta, Chris Wolverton,

Stefan Goedecker, and EKU Gross. Superconductivity in metastable phases of phosphorus-hydride compounds under high pressure. *Phys. Rev. B*, **93**, 020508, (2016).

[88] Andrew Shamp, Tyson Terpstra, Tiange Bi, Zackary Falls, Patrick Avery, and Eva Zurek. Decomposition products of phosphine under pressure: PH_2 stable and superconducting? *J. Am. Chem. Soc.*, **138**, 1884-1892, (2016).

[89] Defang Duan, Fubo Tian, Xiaoli Huang, Da Li, Hongyu Yu, Yunxian Liu, Yanbin Ma, Bingbing Liu, and Tian Cui. Decomposition of solid hydrogen bromide at high pressure. *arXiv preprint arXiv:1504.01196*, (2015).

[90] Miguel Borinaga, P Riego, A Leonardo, Matteo Calandra, Francesco Mauri, Aitor Bergara, and Ion Errea. Anharmonic enhancement of superconductivity in metallic molecular Cmca-4 hydrogen at high pressure: a first-principles study. *Journal of Physics: Condensed Matter*, **28**, 494001, (2016).

[91] Paolo Giannozzi, Stefano Baroni, Nicola Bonini, Matteo Calandra, Roberto Car, Carlo Cavazzoni, Davide Ceresoli, Guido L Chiarotti, Matteo Cococcioni, Ismaila Dabo, Andrea Dal Corso, Stefano de Gironcoli, Stefano Fabris, Guido Fratesi, Ralph Gebauer, Uwe Gerstmann, Christos Gougoussis, Anton Kokalj, Michele Lazzeri, Layla Martin-Samos, Nicola Marzari, Francesco Mauri, Riccardo Mazzarello, Stefano Paolini, Alfredo Pasquarello, Lorenzo Paulatto, Carlo Sbraccia, Sandro Scandolo, Gabriele Sclauzero, Ari P Seitsonen, Alexander Smogunov, Paolo Umari, and Renata M Wentzcovitch. QUANTUM ESPRESSO: a modular and open-source software project for quantum simulations of materials. *Journal of Physics: Condensed Matter*, **21**, 395502, (2009).

[92] P Giannozzi, O Andreussi, T Brumme, O Bunau, M Buongiorno Nardelli, M Calandra, R Car, C Cavazzoni, D Ceresoli, M Cococcioni, N Colonna, I Carnimeo, A Dal Corso, S de Gironcoli, P Delugas, R A DiStasio, A Ferretti, A Floris, G Fratesi, G Fugallo, R Gebauer, U Gerstmann, F Giustino, T Gorni, J Jia, M Kawamura, H-Y Ko, A Kokalj, E KÃŒçÃŒkbenli, M Lazzeri, M Marsili, N Marzari, F Mauri, N L Nguyen, H-V Nguyen, A Otero de-la Roza, L Paulatto, S Poncé, D Rocca, R Sabatini, B Santra, M Schlipf, A P Seitsonen, A Smogunov, I Timrov, T Thonhauser, P Umari, N Vast, X Wu, and S Baroni. Advanced capabilities for materials modelling with quantum ESPRESSO. *Journal of Physics: Condensed Matter*, **29**, 465901, (2017).

[93] John P. Perdew, Kieron Burke, and Matthias Ernzerhof. Generalized Gradient Approximation Made Simple. *Phys. Rev. Lett.*, **77**, 3865-3868, (1996).

[94] M. Methfessel and A. T. Paxton. High-precision sampling for brillouin-zone integration in metals. *Phys. Rev. B*, **40**, 3616-3621, (1989).

[95] Bader R. F. W. *Oxford University Press*, Oxford, (1990).

[96] A.D. Becke and K.E. Edgecombe. A simple measure of electron localization in atomic and molecular systems. *J. Chem. Phys*, **92**, 5397, (1990).

[97] M. Kohout and A. Savin. Atomic shell structure and electron numbers *J. Quant. Chem.*, **60**, 875-882, (1996).

[98] J. Contreras-Garcia and J. M. Recio. Electron delocalization and bond formation under the elf framework. *Theor. Chem. Acc.*, **128**, 411, (2011).

[99] A. Otero de la Roza and Erin R. Johnson and Víctor Luaña Critic2: A program for real-space analysis of quantum chemical interactions in solids. *Computer Physics Communications* , **183**, 1007-1018, (2014)

[100] Miguel Borinaga, Ion Errea, Matteo Calandra, Francesco Mauri, and Aitor Bergara. Anharmonic effects in atomic hydrogen: Superconductivity and lattice dynamical stability. *Phys. Rev. B*, **93**, 174308, (2016).

[101] A. Otero de la Roza and M.A. Blanco and A. Martín Pendás and Víctor Luaña Critic: a new program for the topological analysis of solid-state electron densities. *Computer Physics Communications*, **180**, 157-166, (2009)
